# Droplet-Based Microfluidics as a Platform to Design Food-Grade Delivery Systems Based on the Entrapped Compound Type

**DOI:** 10.3390/foods12183385

**Published:** 2023-09-09

**Authors:** Jhonatan Rafael de Oliveira Bianchi, Lucimara Gaziola de la Torre, Ana Leticia Rodrigues Costa

**Affiliations:** 1 Department of Materials and Bioprocess Engineering, School of Chemical Engineering, University of Campinas, Av. Albert Einstein, 500, Campinas 13083-852, Brazil; j222748@dac.unicamp.br (J.R.d.O.B.); ltorre@unicamp.br (L.G.d.l.T.); 2 Institute of Exact and Technological Sciences, Federal University of Viçosa (UFV), Campus Florestal, Florestal 35690-000, Brazil

**Keywords:** microchannels, droplet, hydrophilic compound, hydrophobic compound, drug delivery, emulsion

## Abstract

Microfluidic technology has emerged as a powerful tool for several applications, including chemistry, physics, biology, and engineering. Due to the laminar regime, droplet-based microfluidics enable the development of diverse delivery systems based on food-grade emulsions, such as multiple emulsions, microgels, microcapsules, solid lipid microparticles, and giant liposomes. Additionally, by precisely manipulating fluids on the low-energy-demand micrometer scale, it becomes possible to control the size, shape, and dispersity of generated droplets, which makes microfluidic emulsification an excellent approach for tailoring delivery system properties based on the nature of the entrapped compounds. Thus, this review points out the most current advances in droplet-based microfluidic processes, which successfully use food-grade emulsions to develop simple and complex delivery systems. In this context, we summarized the principles of droplet-based microfluidics, introducing the most common microdevice geometries, the materials used in the manufacture, and the forces involved in the different droplet-generation processes into the microchannels. Subsequently, the encapsulated compound type, classified as lipophilic or hydrophilic functional compounds, was used as a starting point to present current advances in delivery systems using food-grade emulsions and their assembly using microfluidic technologies. Finally, we discuss the limitations and perspectives of scale-up in droplet-based microfluidic approaches, including the challenges that have limited the transition of microfluidic processes from the lab-scale to the industrial-scale.

## 1. Introduction

Microfluidics is the science and technology that investigates and manipulates fluids in micrometer-scale channels [1]. It is a multidisciplinary technology that involves fundamental concepts from several fields, including chemistry, physics, biology, and engineering [2]. Microfluidics equally allows a diverse array of applications within these fields, including chemical analysis [3], diagnostics [4], microreactors [5], drug scanning [6], and delivery systems [7]. One of the main advantages of microfluidics is its ability to manipulate fluids within micrometer-scale channels. That offers several benefits, such as decreased reagent consumption, precise control over experimental conditions, and the establishment of laminar flow patterns [8].

Droplet-based microfluidics is a branch of microfluidics in which small droplets are generated one-by-one under the effect of balanced forces between immiscible fluid phases, usually oil and water [2,9,10], generated from the dispersion of droplets of one fluid (disperse phase) in another immiscible one (continuous phase), which are called emulsions. Emulsions are present in several cosmetic, pharmaceutical, and food products to improve sensory and functional attributes, provide protection and stability to functional compounds susceptible to oxidation or hydrolysis, and currently control and sustain their release [11,12,13].

In conventional emulsification methods, nonuniform mechanical forces (shear or/and cavitation forces) involved in the droplet breakup lead to emulsions with high-droplet-size polydispersity [14,15]. Polydisperse droplets affect the compound’s release profile, making sustained and controlled delivery a major challenge. Difficulties with localizing compound delivery and generating carriers loaded with multiple therapeutic agents are also limitations of conventional emulsification methods [7]. In addition, the high energy applied in these methods can increase the system’s temperature, making the addition of temperature-sensitive compounds unfeasible [15]. These processes’ low encapsulation and low energy efficiency also result in higher costs for the industry [15,16].

Droplet-based microfluidics provides a novel approach to overcoming these problems through a rational design of delivery systems with precise control of emulsification processes [17,18]. The benefit of manipulating fluids in laminar flow with demanding low-energy, highly monodisperse droplets can be generated into microchannels and used as templates for developing complex multiphase and multicomponent microcarriers, such as multiple emulsions, microgels, microcapsules, solid lipid particles, and giant liposomes [19,20,21,22]. Thus, unlike emulsions produced by conventional methods, droplet-based microfluidic systems allow for the controlled and sustained delivery of multiple therapeutic agents and temperature-sensitive compounds to target sites. 

**Figure 1:**
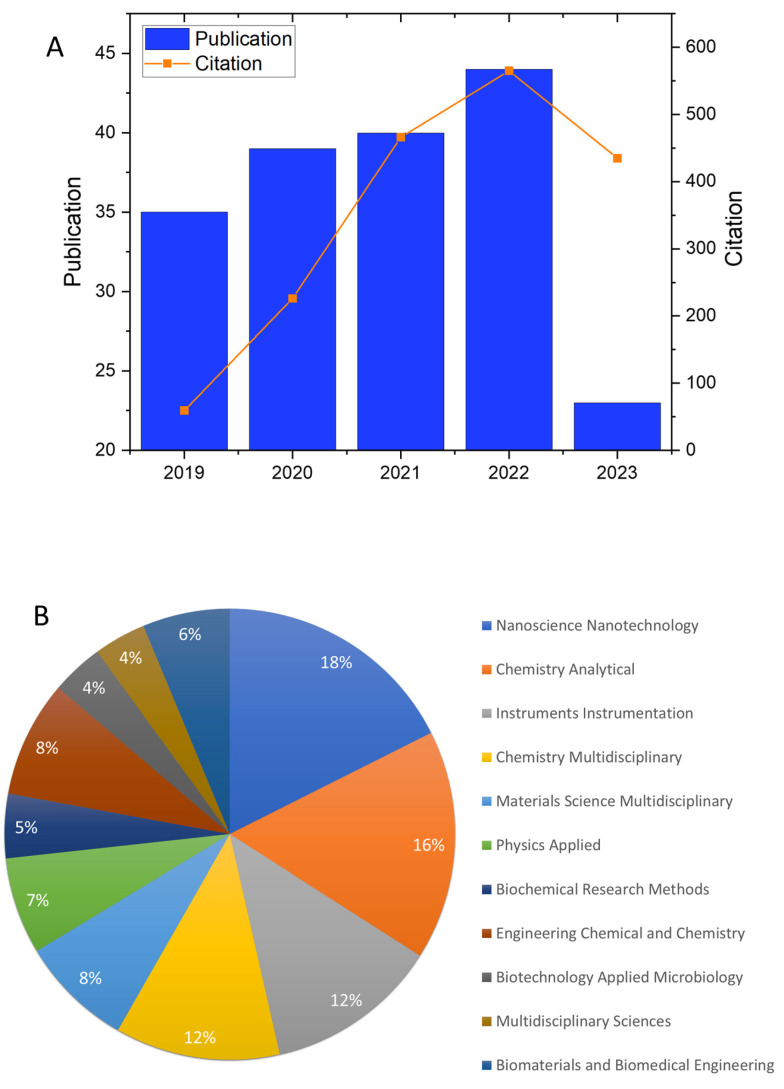
(**A**) Number of publications and citations (2019–2023) on Web of Science using the keywords food-grade, emulsion, droplet microfluidics, and droplet-based microfluidics. (**B**) Percentage of the main knowledge areas with publications related to droplet-based microfluidic systems. All data were collected from the Web of Science database.

Safety-related aspects have driven the development of new strategies that aim to minimize toxicity and increase the specificity of delivery systems through more efficient processes, such as microfluidics and more biocompatible materials. Biocompatible carrier materials can be used as delivery systems in various applications, including regenerative medicine, disease treatment and prevention, and food and cosmetics industries [16,23,24,25]. In general, desirable characteristics in delivery systems are associated with their loading capacity and retention, the delivery mechanism, and the type of protection provided to the functional compound, as well as the commercial viability and safety aspects of the carrier material [12]. Synthetic materials have been widely explored as carriers in microfluidic-assembled delivery systems, while few are food-grade [26]. However, the assembly of food-grade delivery systems using microfluidic approaches has been the subject of interest in many studies since several food-grade materials emerged as viable options for this proposal [27,28,29,30]. As shown in Figure 1A, over the past 5 years, there has been an increase in the number of citations and publications on food-grade emulsion-based delivery systems obtained by microfluidic techniques (keywords: food-grade, emulsion, droplet microfluidics, and droplet-based microfluidics). Beyond its applications in the food industry, food-grade emulsion can be utilized in various other fields, as demonstrated in Figure 1B. For example, in oral administration applications, the delivery system must be produced entirely from food-grade ingredients using processing operations that have regulatory approval. Food-grade ingredients, such as carbohydrates, proteins, and lipids, are typically inexpensive, available, biodegradable, and highly biocompatible [31]. Therefore, some studies already report the development of food-grade systems for delivery by other routes, such as local injection [32]. 

Other review articles have already explored the challenges and applications of droplet microfluidics for encapsulating molecules. For instance, Zhao-Miao, Yang, and Pang (2017) [33] examined the relationship between droplet structures and microdevice design. Other works have focused on fabricating specific structures for encapsulating nutraceutical bioactives [34]. Additionally, in 2021, Schroen et al. [35] wrote a review to show the application of droplet microfluidics in biotechnology and microbiology. It is currently known that microfluidic techniques also allowed, through the choice of ingredients and the emulsification process parameters, to precisely modulate the properties of the carrier systems to suit the type of entrapped functional compound and its final application. It is of great interest to design delivery systems suited to the specific encapsulated compound to improve its bioavailability, therapeutic efficacy, and sustained delivery to target sites. Accordingly, this review points out the most current advances in droplet-based microfluidic processes, in which food-grade materials were successfully utilized to develop several delivery systems, including single/multiple emulsions, microgels, microcapsules, solid lipid microcapsules, and giant liposomes. In addition, the entrapped compound type, classified as lipophilic or hydrophilic functional compound, was used as a starting point to present current advances in delivery systems based on food-grade emulsions and assembly using microfluidic technologies.

**Figure 2:**
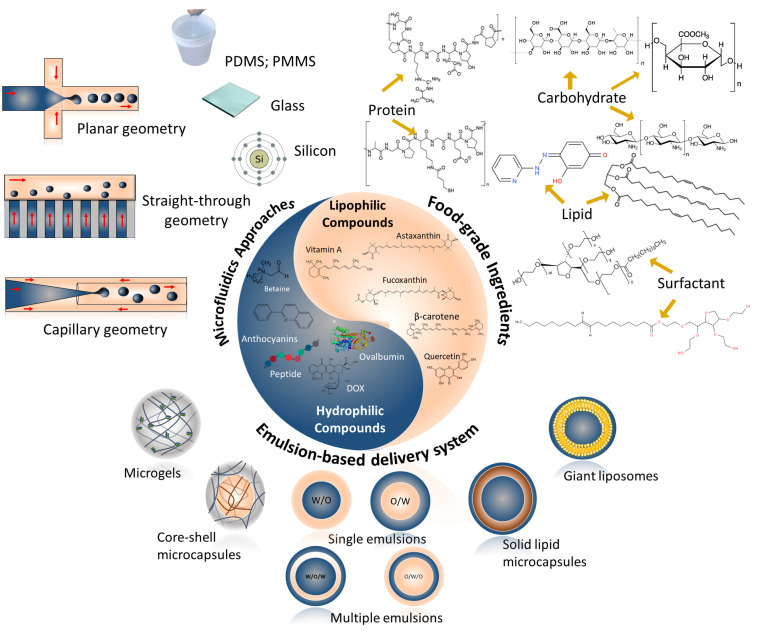
Schematic representation of the main topics on microfluidic assembly for the design of food-grade emulsion-based delivery systems with a focus on the entrapped compound type.

Figure 2 is a schematic representation of the main topics covered in this review on microfluidic assembly for the design of food-grade emulsion-based delivery systems with a focus on the entrapped compound type, which are: (i) microfluidic approaches, (ii) food-grade ingredients, and (iii) emulsion-based delivery systems.

## 2. Principles of Droplet-Based Microfluidics

Understanding the fluid dynamics behind microfluidic devices is essential for successfully developing droplet-based systems. Droplet generation by microfluidic techniques introduces nonlinear laws into the otherwise linear Stokes flows related to the complexity of fluid-dynamic events during droplet formation and the breakup into the microchannels [36]. Additionally, since liquids flow in tiny channels, surface effects become more important than the macroscale [37]. Thus, when two mutually immiscible fluids flowing in the microchannels come into contact, the forces (e.g., interfacial, viscous, or inertial) involved in the droplet-formation process act immediately on the fluids [37,38]. These droplet-generation methods, called passive, positive-displacement pumps (syringes pumps), control the fluid flow into the channels. The droplet formation and breakup are strongly affected by the microchannel properties (such as material, design, wettability, and roughness) and fluid properties (such as density, viscosity, emulsifier type, and concentration) [21,30].

Several techniques (soft lithography, micromachining, and laser ablation) and materials (silicon, glass, and polydimethylsiloxane-PDMS) manufacture microfluidic devices with different geometries. In general, the choice of microdevice type will depend on the desired droplet-based system. Droplet-generation devices are based on shear-induced geometries and interfacial-tension-induced ones. The droplet-formation process in shear-induced geometries occurs under the effect of balanced forces (interfacial, viscous, and inertial), while in interfacial-tension-induced geometries, the droplet is generated spontaneously as only a function of the interfacial tension between immiscible fluid phases. The most commonly used geometries for obtaining food-grade emulsions through interfacial-tension- and shear-induced geometries are oblong straight-through geometry and capillary assemblies/planar geometries, respectively [39,40,41]. In addition, due to the hydrophilic nature of silica and glass, these materials are mainly used to produce oil droplets dispersed in water (O/W emulsions), whereas PDMS, a hydrophobic polymer, is primarily used to disperse water droplets in oil (W/O emulsions). However, to design more complex droplet-based systems, some strategies for modifying the surface of microchannels obtained from these materials are proposed, such as layer-by-layer deposition and covalent surface modification via graft photopolymerization [25,41,42].

### 2.1. Straight-Trough Geometry

**Figure 3:**
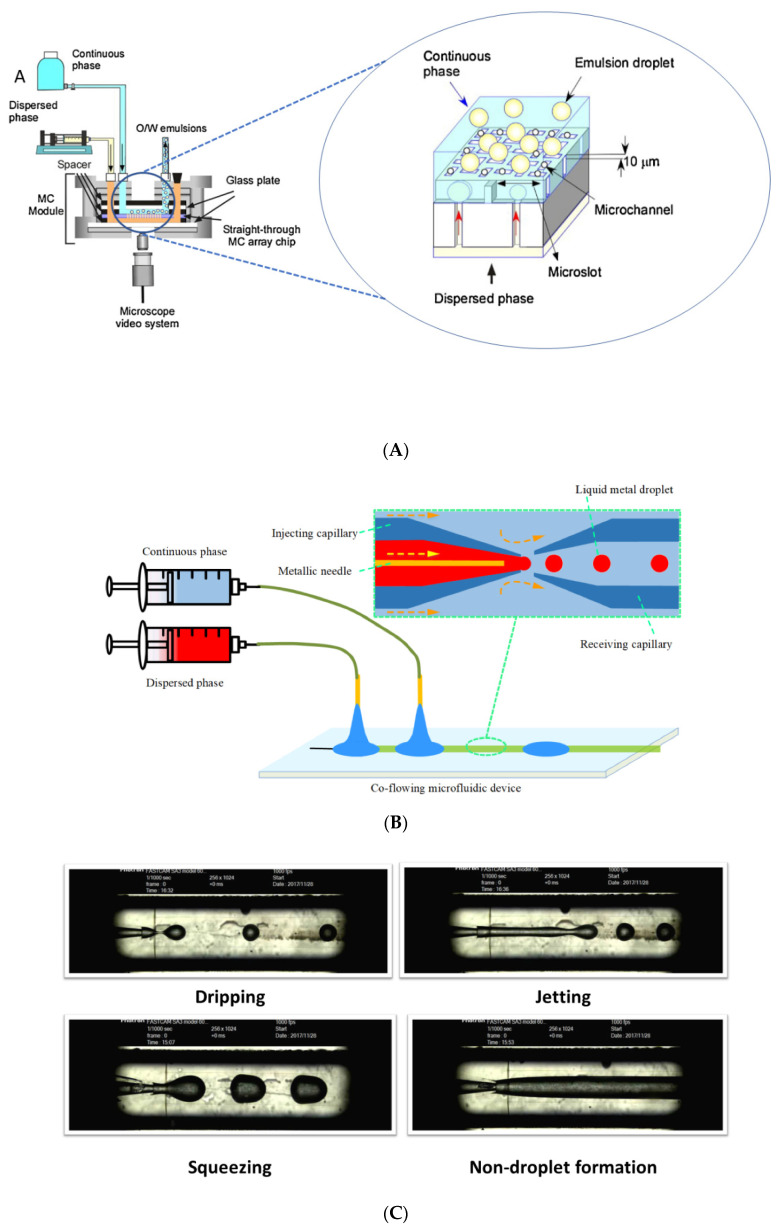
(**A**) Graphical representation of a straight-through microfluidic device setup and droplet-generation representation through oblong straight-through geometry (adapted from: [45]) (**B**) Graphical representation of a shear-induced microfluidic device setup. (**C**) Droplet formation regimes of shear-induced geometries [46].

Currently, most microdevices based on the oblong straight-through geometry used to produce emulsion-based carrier systems are commercial silicon chips microfabricated using the repeated deep-reactive-ion etching (DRIE) process and photolithography [16,43,44]. The silicon chip is tightly held between two rubber spacers in the microfluidic module (MC module), and the droplet-formation behavior is directly observed on the microscope video system (Figure 3A). The dispersed phase is forced to pass through the microchannel in a controlled flux, provided by syringe pumps, to an area previously filled with a continuous phase, which is supplied from a reservoir elevated to the chip (Figure 3) [16,43]. The droplet-generation process occurs when the fluid interface near the tip of the channel is forced to elongate during the droplet growth across the channel (highlighted in Figure 3A). Then, the force to restore the interface tension gradient of the elongated interface spontaneously shears the droplet from the tip of the channel [39].

### 2.2. Shear-Induced Geometries Microfluidics Device

Shear-induced geometries are based on planar geometries and capillary assemblies [41]. Capillary microfluidic devices consist of coaxial assemblies of square and cylindrical glass capillaries [47]. Typically, the cylindrical capillary is inserted inside a square with a large diameter and placed on a glass slide. Hypodermic needles attached to the devices allow fluid injection into the microchannel through plastic tubing connected to syringe pumps [46]. Usually, the dispersed phase flowing inside the cylindrical capillary is constricted by the continuous phase that flows between the capillaries to generate single-emulsion droplets (Figure 2B). Despite all the advantages of producing emulsion-based carrier systems using capillary devices (Table 1), the intensive labor involved in manufacturing and aligning the tubes [47] drives the use of alternative devices.

Planar devices are microchannels with rectangular cross-sections produced primarily with flexible PDMS by the soft-lithography technique [48]. In this technique, a master produced by photolithography is used to create a mold. PDMS or another elastomer precursor with a curing agent is poured into the mold and cured. The device is then peeled off and closed with a glass or PDMS coverslip. This easy and simple method of designing and manufacturing planar devices overcomes some of the challenges associated with capillary ones, allowing for more elaborate design configurations [49,50]. The configuration of planar devices is defined according to the junction type between the microchannels, generally classified as T-, Y-, and cross-junctions. Unlike interfacial-tension-induced geometry, droplet formation in planar geometries is mainly a result of the shear force promoted by the continuous phase on the dispersed phase. The continuous phase flowing on either side of the dispersed-phase stream at the cross-junction causes higher shear than at the T- or Y-junctions. This configuration, known as hydrodynamic flow-focusing, has been widely utilized for producing emulsion-based carrier systems because it generates small and uniform droplets [41,51,52].

### 2.3. Droplet-Formation Regimes

In both shear-induced geometries, process parameters and fluid properties regulate the velocity of droplet detachment, which defines the three droplet-formation regimes: dripping, squeezing, or jetting (Figure 3C) [53,54]. The jetting regime occurs when forces related to the interfacial tension dominate the continuous-phase shear forces. Thus, the dispersed phase forms a long neck along the main channel, and droplet breakup occurs downstream based on the Rayleigh–Plateau uncertainty principle [55,56]. In the squeezing regime, the dispersed phase accumulates for a short period, occupying almost the entire transversal area of the main channel until the droplet pinch-off is triggered by the pressure differential behind and in front of a confined extension of the fluid interface [46,57]. In the dripping regime, the viscous dragging forces quickly overcome the interfacial ones. Thus, the dispersed phase is broken before filling the entire cross-section area of the main channel, generating droplets smaller than those produced in the squeezing regime. The compound’s more efficient and controlled release to the target site is usually achieved with smaller droplets; therefore, the dripping regime is desired in most droplet-based microfluidic processes [58]. No droplets are formed if the velocity of the dispersed phase is greater than that of the continuous phase. Under these flow conditions, highly viscous dispersed phases can hamper the action of shear forces, preventing droplet formation [59].

Dimensionless numbers, such as Reynolds, Weber, and Capillary, play a significant role in elucidating the influence of dominant forces during droplet generation in microfluidic devices and flow patterns. The Reynolds number (Re) is often used to predict the flow regime (laminar or turbulent), describing the relation between inertial forces and viscous ones (Equation (1)) [60]. The laminar regime is prevalent in microfluidics due to the low velocities of fluids within microscale channels [61]. The immiscible phases are typically two fluids with distinct properties, resulting in varying flow velocities. This complicates operating conditions, as more than one infusion pump is needed to control the flow. Additionally, when two immiscible fluids flow in the microchannels, the interfacial tension becomes important, and other dimensionless numbers can better describe the fluid behavior in microfluidic devices [60].

The Capillary number (Ca) is often used to parametrize droplet-breaking processes when viscous effects are more important than inertial ones, while the Weber number (We) is used when inertial stresses dominate viscous ones [36]. The Ca (Equation (2)) and We (Equation (3)) numbers relate the viscous and inertial stresses in the flow to the interfacial stresses, respectively.

(1) $$Re=\frac{\rho ul}{\mu }$$

(2) $$Ca=\frac{\mu U}{\gamma }\;$$

(3) $$We=\frac{\rho U^{2}l}{\gamma }\;$$

where *μ* is the dynamic viscosity (Pa·s), *U* is the characteristic velocity of the fluid (m/s), *ρ* is the density (kg/m^3^), *l* is the characteristic dimension of the channel (m), and *γ* is the interfacial tension (J/m^2^).

Critical transition values between droplet-formation regimes are not standardized in the literature, as the microchannels’ geometry and the fluids’ actual flow rates can strongly influence these numbers. However, microfluidic droplet formations are known to have typical Ca number values ranging from ~10^−3^ to 10^1^ for flow rates accessible using syringe pumps [62]. In addition, as the Ca number increases, a gradual transition from squeezing to dripping and jetting is observed [62].

The advantages and disadvantages of planar, capillary, and straight-through microfluidic devices are presented in Table 1. Each can enhance or limit the application of the microfluidic device in developing emulsion-based delivery systems, including in large-scale processes. Commercial devices are an important part of the recent advances in droplet-based microfluidic processes, as they have been used successfully to design carrier systems based on food-grade emulsions. Table S1 (Supplementary Material) summarizes the main technical specifications of commercial devices, as knowing them can expand their use in a range of applications (i.e., food, biology, and chemical) and encourage further studies related to new processes and products.

## 3. Microfluidic Assembly of Food-Grade Delivery Systems Based on Entrapped Compound Type 

### 3.1. Lipophilic Functional Compounds

The food, pharmaceutical, cosmetic, and medical industries require the delivery of a variety of lipophilic functional components, including bioactive lipids, nutraceuticals, therapeutic agents, and drugs. However, many of these compounds are prone to degradation and crystallization when exposed to environmental conditions. They also often exhibit poor water solubility, unpleasant odor, and low bioavailability [12,63,64]. Emulsion-based delivery systems have been reported to address most of these drawbacks, including enhanced solubility and chemical stability [65,66,67].

#### 3.1.1. Single Oil–Water (O/W) Emulsion

The single oil-in-water (O/W) emulsions are the simplest delivery systems for encapsulating lipophilic functional compounds. These systems ensure protection and target delivery by dispersing oil droplets containing the lipophilic compound in an aqueous phase containing an emulsifier or surfactant [58,68,69]. Emulsifiers are amphiphilic molecules that act at the oil–water interface, reducing the tension between the phases, favoring the droplet breakup, and preventing the recoalescence of the droplets during the emulsification process [70]. The type and concentration of the emulsifiers, as well as the droplet size polydispersity, are the main factors that govern the kinetic stability of these systems against the main destabilization mechanisms (i.e., flocculation, coalescence, creaming, and Ostwald ripening) [12]. There are a significant number of emulsifiers classified as food-grade ingredients, including low-molecular-weight surfactants (polyoxyethylene sorbitan fatty acid esters (Tweens), polyglycerol polyricinoleate (PGPR), and sorbitan fatty acid esters (Spans)), phospholipids (soybean lecithin), and biopolymers (proteins and carbohydrates) [68,71].

Khalid et al. (2016) [45] investigated the effects of the emulsifier type, bovine serum albumin (BSA), and polyoxyethylene (20) sorbitan monolaurate (Tween 20) on droplet-formation characteristics and the stability of emulsions encapsulating quercetin produced in straight-through microfluidic devices. Both emulsifying molecules have nonattractive interaction with the chip surface; thus, uniform-sized droplets could be generated regardless of the type of emulsifier. However, more stable droplet generation and smaller droplet size were observed with Tween 20 (nonionic emulsifier) compared to BSA (protein emulsifier). After optimizing microfluidic process parameters (*J_d_* = 20 L/m^2^ h^1^ and *Q_c_* = 250 mL/h) and ingredient formulations (1% *w*/*w* Tween 20 and 0.4 mg/mL quercetin in MCT oil), the delivery systems showed an encapsulation efficiency superior to conventional emulsification methods, exceeding 70% after 30 days of storage at 4 and 25 °C. In a similar study, Khalid, Shu, et al. (2017) [43] showed that the purity of the lipophilic compound could also influence the droplet-generation process in straight-through microfluidic devices. Typically, low-purity commercial compounds have stabilizing ingredients that disfavor emulsification. In these cases, optimized process conditions and suitable emulsifiers are key factors for successful encapsulation. Different emulsifiers (1% *w*/*w* sodium dodecyl sulfate (SDS), decaglycerol monooleate (MO-7S), decaglycerol monolaurate (ML-750), modified lecithin (ML), and sodium caseinate (Na-Cs)) with different stabilizing mechanisms were used to stabilize O/W emulsions encapsulating astaxanthin (AXT) extracts in two purity degrees, Zanthin^®^ (ZA, purity 10%) and AstaReal^®^ (AR, purity 20%). All emulsifiers, except Na-Cs, promoted the production of uniform droplets of MCT oil with the ZA extract into the microchannels. The same behavior was observed during the generation of oil droplets with the AR extract stabilized by polyglycerol fatty acid esters (ML-750 and MO-7S) and the ionic emulsifier (SDS). In contrast, broad size-distribution curves confirmed the unstable generation of oil droplets with the AR extract stabilized by protein-based emulsifiers (Na-Cs and ML). These results showed that the chemical composition of the lipophilic compound could directly affect the ability of the emulsifier to reduce the interfacial tension between the oil–water phases, which may or may not favor the process of forming stable droplets in straight-through microfluidic devices.

Straight-through microfluidic devices were also used to assess the effect of different concentrations of two different lipophilic compounds, γ-oz and β-st, on the droplet-formation characteristics of emulsions [45]. At low concentrations (0.5–1% (*w*/*w*) each) of γ-oz and β-st and *Q_d_* between 1 and 5 mL/h, small droplets (diameters between 26.5 and 28.5 μm) could be formed uniformly with no signal of wetting by the dispersed phase on the chip surfaces. Additionally, very small Ca numbers (1.0 × 10^−3^–1.4 × 10^−3^) indicated a smooth droplet generation into the microchannels without the influence of different flow rates on the droplet size. On the other hand, at high concentrations of γ-oz and β-st (1.0–4% (*w*/*w*) each), an increase in droplet size and polydispersity was observed, which may be associated with the crystallization of the lipophilic compounds in the dispersed phase. The emulsions formulated with γ-oz and β-st (1% *w*/*w* Tween 20 and *Q_d_* = 2 mL/h) maintained the encapsulation efficiency of more than 80% during 30 days of storage at room and refrigerated temperatures. Additionally, the γ-oz and β-st retention values in the O/W emulsions (14 μg/mL and 53 μg/mL, respectively) correlated well with the recommended daily intake values.

A recent study compared fucoxanthin’s chemical stability and bioaccessibility during the in vitro digestion of O/W emulsions produced by straight-through microchannel emulsification and high-pressure homogenization [72]. The chemical stability of fucoxanthin in emulsions produced by microfluidics was significantly higher than those produced by high-pressure homogenization. In contrast, the free fatty acids released and fucoxanthin bioaccessibility in emulsions using microfluidics (around 10%) were significantly lower than using a high-pressure homogenizer (around 60%). The high-energy-emulsification process caused the degradation of fucoxanthin but led to significantly smaller droplet generation than microfluidics (0.14 μm and 33.7 μm, respectively). Large oil droplets reduced the triacylglycerol molecules exposed to the lipase action; thus, only a small amount of fucoxanthin could be released from emulsions produced by straight-through microchannel emulsification.

As lipid digestion is an interfacial process, the oil droplet size and the nature of carrier ingredients play an important role in the lipid hydrolysis (lipolysis) rate of O/W emulsions [68,73,74]. Most food-grade emulsions are produced with medium-chain triglycerides (MCT) or long-chain triglycerides (LCT), such as soybean oil, rapeseed oil, and sunflower oil [44,72,75,76]. The LCT oil is formed by unsaturated fatty acids, which makes its molecular structure more complex and bent, whereas the MCT oil is saturated with a linear molecular structure [69]. These structural differences affect the hydrophobicity, solubility of the lipophilic compound, and lipolysis rate of O/W emulsions during digestion. Long-chain free fatty acids accumulate at the oil-in-water interface, whereas medium-chain ones tend to move towards the aqueous phase due to their lower hydrophobicity [77]. Thus, LCT systems provide lipophilic compounds with higher bioaccessibility and protection during digestion than MCT systems [69].

Furthermore, since many lipophilic compounds must be absorbed in the intestine to promote health benefits, they must pass the gastric digestion step intact, resisting the stomach acid pH [78]. Some gelling biopolymers, such as gelatin, alginate, kappa-carrageenan, and pectin, resist gastric conditions and can protect the bioactive until it reaches the small intestine to be absorbed [29,78,79,80]. These biopolymers can form three-dimensional (3D) networks composed of crosslinked hydrophilic chains that allow the encapsulation of hydrophilic compounds. Lipophilic compounds can be encapsulated by creating a core surrounded by the polymer matrix [44], making emulsions ideal templates for obtaining these systems. J. Zhang, Zhang et al. (2021) [81] designed a simple method to prepare an O/W emulsion encapsulating vitamin A using a microscale 3D-printed microfluidic device. Sodium alginate, gelatin, and ethylenediaminetetraacetic acid calcium disodium salt hydrate (EDTA-Ca) were used as the continuous phase, while vitamin A mixed with tert-butyl hydroquinone (ratio of 4:1) was used as the dispersed phase. The emulsion droplets were collected in an acid environment similar to the gastric fluid to generate microgels, since the acid caused calcium ion (Ca^2+^) leakage from the EDTA-Ca and crosslinking with sodium alginate. The low vitamin A concentration in the simulated gastric fluid (about 20%) indicated that the microgels were stable, preventing the release of the bioactive. However, the polymeric matrix of the microgels was destroyed in simulated intestinal fluid, allowing the release of vitamin A (around 75%) within 2.5 h.

Unlike highly hydrophilic biopolymers, synthetic polymers can be built from different monomers that, depending on original features and proportions in the polymer chain, define the chemical and physical properties of the synthesized material [82]. Some synthetic polymers have already been classified as food-grade, such as poly-lactic-co-glycolic acid (PLGA), polymethyl methacrylate (PMMA), polyethyleneimine (PEI), poly (vinyl alcohol) (PVA), and polyethylene glycol (PEG) [26,83,84]. PLGA is the most commonly used synthetic polymer for microfluidic assembly as a carrier material [27,28,85]. It is a copolymer comprising PGA (polyglycolic acid) and PLA (polylactic acid) that decomposes into nontoxic byproducts through the Krebs cycle. Additionally, by changing the proportions of PGA (hydrophobic compound) and PLA (hydrophilic compound), the degree of hydrophilicity and lipophilicity can be modulated to encapsulate both lipophilic or hydrophilic drugs [86,87].

Finasteride, a highly lipophilic compound, was encapsulated in PLGA microspheres using a microfluidic chip containing seven parallel microchannels (Inventage Lab Inc. Precision Particle Fabrication). PLGA 5050A (acid-terminated with a lactide/glycolide ratio of 50/50) or PLGA 7525A (acid-terminated with a lactide/glycolide ratio of 75/25) were dissolved in dichloromethane (DCM) followed by the addition of finasteride (28 mg) to compose the dispersed phase. At the same time, the aqueous solution of PVA (0.25% *w*/*v*) was used as the continuous phase [27,28]. The dispersed and continuous phases were inserted into the microfluidic chip with a pressure of 1100 mbar and 2200 mbar, respectively. Finasteride-added PLGA droplets generated within the channels were polymerized after DC evaporation, lyophilized, and injected subcutaneously into healthy male beagle dogs to assess their drug release and pharmacokinetics in vivo. The microspheres generated by parallelized microchannels showed high encapsulation efficiency (96.5–101.2%) and particle sizes within the recommended range for injectable dosage forms (all smaller than 50 μm). Microspheres produced with PLGA 7502A (75:25 copolymer) exhibited a lower initial drug release and greater extended release (about 1 month) in beagle dogs than microspheres based on PLGA 5002A (50:50 copolymer). Additionally, the in vivo drug-release profile was proportionally related to the amount of drug loading.

PLGA has also been used to synthesize drug-carrying nanoparticles using the nanoprecipitation method by droplet-based microfluidic approaches [85,88,89]. Nanoparticles are widely studied as potential strategies to enhance functional compounds’ solubility, bioavailability, circulation time, and delivery efficiency due to their high specific surface area [90,91]. Polymeric nanoparticles form within the microchannels when a droplet of the polymer, dissolved in an organic solvent, reduces its solubility upon mixing with the water-miscible organic solvent and an aqueous solution [85]. However, the mixing efficiency may be a limiting factor in using the nanoprecipitation method in microchannels due to the laminar flow regime of the fluids. Staggered herringbone mixers (SHMs) are passive mixers inserted into channel walls to destabilize the laminar flow and increase the mixing efficiency [92]. Rutin-loaded PLGA nanoparticles were generated in microfluidic devices with SHMs using rutin (10 mg/mL) in methanol, and PLGA (14.9 mg/mL) in acetonitrile as the dispersed phase (or organic solvent phase) and PVA (1% *w*/*v*) as the continuous phase (or aqueous phase) [85]. The optimal formulation of rutin-loaded PLGA nanoparticles prepared by the microfluidics method showed an entrapment efficiency of 34 ± 1%, a size of 123 ± 4 nm, and a drug loading of 0.015 ± 0.001%. Rutin-loaded PLGA nanoparticles produced in the microfluidic devices exhibited a faster release of rutin with a higher burst release than those produced in the bulk method. Similar studies applied droplet-based microfluidics using glass and stainless-steel metal devices to obtain nanoparticles of itraconazole and fenofibrate by nanoprecipitation methods. Metal cross-junction channels provided a good mixing environment for generating smaller, more uniformly sized itraconazole particles. In contrast, glass microfluidic devices provided an inert and stable platform for creating highly monodisperse fenofibrate nanoparticles [88,89].

#### 3.1.2. Double Water–Oil–Water (W/O/W) Emulsion 

**Figure 4:**
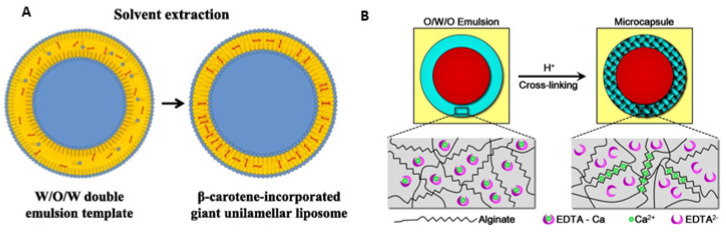
(**A**) Schematic illustration of obtaining alginate core microcapsules from the O/W/O emulsion generated in glass microfluidic devices. After collecting the O/W/O emulsions, the aqueous middle phase was gelled with calcium ions released from the water-soluble calcium complex after mixing with an acidified oil solution [76]. (**B**) Diagram of the organic solvent extraction process to form giant unilamellar liposomes from the W/O/W emulsion templates generated in glass microfluidic devices [93].

The high manipulating-fluids control provided by droplet-based microfluidic devices at the laminar regime allows the design of more elaborate structures using double or multiple emulsions as templates, such as oil-in-water-in-oil (O/W/O) and water-in-oil-in-water (W/O/W) [41]. For example, a glass capillary microfluidic device with two emulsion generators and one adjusting unit was designed to prepare O/W/O double emulsions [76]. From the O/W/O emulsion templates, alginate core–shell microcapsules were produced by gelling the middle aqueous phase with calcium ions—released from the water-soluble calcium complex after mixing with acidified oil solution (Figure 4A). Alginate shells had their thickness little affected by the alginate concentration, and their strength was improved by postcrosslinking in polyetherimide, calcium chloride (CaCl_2_), or chitosan solution. The proposed microfluidic approach allowed the precise control of the proportion between different oil droplets (thyme and lavender essential oils) and the number of oil cores in alginate microcapsules by manipulating flow rates in the microfluidic device [76]. In another study, a similar glass microfluidic device was manufactured for producing giant unilamellar liposomes from W/O/W emulsion templates using low-cost, food-grade phospholipids (soybean lecithin powder), and FDA-approved toxicological class III solvents (ethyl acetate and pentane) [93]. Giant unilamellar liposomes are defined as aqueous volumes surrounded by layers of phospholipid molecules. Thus, the middle oil phase consisted of a mixture of soybean lecithin (0.5% *w*/*v*) and β-carotene (0.125% *w*/*v*) dissolved in different organic solvent mixtures.

In comparison, the innermost aqueous phase and the continuous phase contained PVA (1% *w*/*v*) mixed with dextran (9% *w*/*v*) and only PVA (10% *w*/*v*), respectively. After the double emulsion was collected, giant liposomes were generated by dewetting and evaporating the organic solvents in the middle oil phase (Figure 4B). The giant unilamellar liposomes loaded with β-carotene presented diameters varying between 100 μm and 180 μm and exhibited stability for approximately 7 days. Additionally, the presence of β-carotene within the oil shell did not significantly affect the liposome stability, mean diameter, and coefficient of variation compared to those without β-carotene [93]. Thus, all the microfluidic approaches described for producing high-performance delivery systems from food-grade emulsions have the potential to be useful in many applications for protecting, controlling, and sustaining lipophilic functional compounds.

### 3.2. Hydrophilic Functional Compounds

Many pharmaceutical, cosmetic, and food industries require delivery systems for hydrophilic functional compounds, including vitamins, enzymes, proteins, bioactive peptides, and drugs. These applications include the need to mask the bitter taste of drugs and minimize their side effects [94,95], improve the bioavailability of functional molecules, and control–sustain their release at target sites [24,96,97], in flavors, and in water-soluble colors during storage [44]. While lipophilic functional compounds can be carried in O/W emulsions, hydrophilic ones are more adequately protected in water-in-oil (W/O) systems. Additionally, with the addition of gelling polymers in the dispersed phase, a more efficient protection system can be achieved by gelling the emulsion droplets [14,25]. The process of gelling emulsion droplets in microfluidic approaches occurs in two steps. In the emulsification step, the droplet is generated in the microchannels (Section 2), followed by the gelation step, which is the solidification of the emulsion droplets induced by a chemical or physical crosslinking agent [98]. The main gelation methods applied in droplet-based microfluidics (e.g., external, internal, coalescence-induced gelation, and in situ mixing) differ among them by the location of the biopolymer and crosslinking agent in the different phases, and by the triggering mechanism for gelation, where external and internal gelation are the most popular methods. In the first one, crosslinking agent ions diffuse to the droplet interface from the continuous phase or a bath outside the channel. In the second, the biopolymer and the crosslinking agent in the inactive form are inserted together into the channel; the release of ions for gelling is triggered by an additional substance dispersed in the continuous phase (e.g., acetic acid) [14,98].

#### 3.2.1. Single Oil–Water (O/W) Emulsion 

As aforementioned, the 3D network formed by hydrophilic polymers allows the encapsulation of hydrophilic compounds in the polymer matrix. Natural biopolymers derived from animals (e.g., gelatin and chitosan), plants (e.g., pectin), algae (e.g., alginate), and microorganisms (e.g., gellan gum) have become important carrier materials motivated by their excellent biocompatibility, reproducibility, biodegradability, and ability to form gels easily [99]. Ogończyk et al. (2011) [100] produced pectin microgels from W/O emulsions using flow-focusing microfluidic devices. The pectin droplets (0.5–1% *w*/*w*) were solidified by the external gelation method, in which hydrogen and Ca^2+^ were delivered from the continuous phase composed of rapeseed oil, acetic acid (1–10% *w*/*w*), and calcium carbonate (CaCO_3_; 0.05–2% *w*/*w*). This method allowed the encapsulation of gold nanoparticles in pectin microgels and the control of their release rate. Gellan gum microgels incorporated with *jabuticaba* extract, a fruit rich in anthocyanins, were also produced from W/O emulsions by the external gelation method using capillary microfluidic devices [25]. The emulsion droplets generated by the dripping regime were solidified into gellan microgels induced by Ca^2+^ present in the continuous phase (composed of soybean oil, PGPR (4% *w*/*w*), and calcium acetate (1% *w*/*w*)). However, *jabuticaba*-extract-loaded gellan microgels (0.2% *w*/*w*) showed an irregular structure, a flocculated state with an elliptical shape, and low stability, which were mainly associated with the osmotic pressure difference during the storage and the low-gellan-gum concentrations. The flow of gellan gum at high concentrations (i.e., high viscosity fluid) into the microchannels was a process limitation, since Ca^2+^ in the *jabuticaba* extract triggered gellan gelation, which resulted in an uneasy-flowing material before the microchannel inlet [25]. In general, microgels produced from natural polymers have a low mechanical performance. However, strategies associated with the chemical modification and blending of biopolymers to form hybrid or layer-by-layer hydrogels can overcome this limitation once each polymer’s physical and chemical advantages are integrated [101].

L. Yu et al. (2019) [102] combined the internal and external gelation methods to control the morphology of protein–core alginate–shell microgels. In their PDMS microfluidic devices, protein ovalbumin aqueous solution was coflowed with an alginate solution (2% *w*/*v*) containing CaCO_3_ (200 mM) to create a core–shell stream. This stream was further sheared off by the continuous phase, which consisted of mineral oil containing Span 80 (3% *w*/*w*). In general, mineral oil can be safely used in food, and trace amounts remaining would not pose a safety concern [102]. The internal gelation process was started by releasing calcium ions from CaCO3 within the alginate shell, which was achieved by introducing an additional continuous phase composed of mineral oil with Span 80 (3% *w*/*w*) and acetic acid (0.2% *v*/*v*). The external gelation process was completed in a collecting bath containing CaCl_2_ (0.27 M) aqueous solution outside the device, ensuring the microgels’ spherical structure. Furthermore, two approaches were applied to improve the retention of the model protein ovalbumin. In the first one, the alginate microgels were coated with a layer of oppositely charged polymer (poly(ethyleneimine) (PEI) or chitosan). In the second, small particles (inulin microparticles adjuvant) were added inside the water core to block the pores of the polymeric network. The percentage of encapsulation efficiency and protein release of the PEI-coated alginate microgels were 88% and 62% (at 24 h), respectively. In contrast, for the chitosan-coated alginate microgels, these values were 80% and 100% (at 48 h), respectively. The highest encapsulation efficiency and sustained-controlled protein delivery were achieved when the two strategies were combined; PEI-coated ovalbumin-delta inulin-encapsulated alginate microgels achieved up to 90% encapsulation efficiency and 20% protein release after 7 days.

Some proteins with intracellular activity have significant potential to treat Crohn’s disease and ulcerative colitis [24]. In these applications, ensuring protein protection and achieving controlled-sustained delivery to the specific target is essential, especially when delivering protein therapeutics via the oral route. However, these proteins must still be internalized into cells and penetrate natural mucus to exert their therapeutic functions [24]. Compared to soluble proteins, protein nanoparticles have been shown to have more capacity to reach inflamed tissue, penetrate the mucosa, and increase cellular internalization [103,104]. Using a PDMS microfluidic device, Ling et al. (2019) [24] produced alginate microgels encapsulating protein (AvrA enzyme) from W/O emulsions by the external gelation method. Alginate droplets were gelled in a collecting bath containing CaCl_2_ and simultaneously coated with chitosan. Chitosan-coated alginate microgels protected and retained protein activity against harsh gastric conditions in vitro, whereas its release was induced only in simulated intestinal fluid. In addition, oral administration of protein nanoparticles encapsulated into alginate/chitosan microgels reduced clinical symptoms and histological inflammation scores in a murine dextran sulfate sodium (DSS)-induced colitis pre/cotreatment model.

Encapsulation of multifunctional compounds in oil-droplet-templated microgels is also a fascinating strategy for medical and pharmaceutical applications, especially in multidrug treatments with high frequencies of administration [105]. Mineral-oil droplets added with quercetin nanoparticles and retinyl palmitate were generated inside the PDMS microfluidic devices in an aqueous phase composed of pectin (1 wt%) and Tween 80 (1 wt%). An extra continuous phase composed of water, ethanol (40 wt%), and calcium chloride (1 wt%) was mixed with the O/W emulsion to solidify pectin on the oil droplets’ surface based on the pectin’s precipitating properties in ethanol and its ionic crosslinking with calcium ions. By changing the flow rate of the phases, the oil core and the shape of the pectin shell were easily controllable. Additionally, core–shell microgels could protect quercetin nanoparticles and retinyl palmitate from degradation and oxidation through exposure to water and oxygen [105].

#### 3.2.2. Double Oil–Water–Oil (O/W/O) Emulsion

As with lipophilic compounds, designing more elaborate structures using double emulsions as templates may also be suitable for encapsulating hydrophilic functional compounds [11,44,75,97,106]. Pagano et al. (2018) [44] described the encapsulation of three different sources of betanin, E162 (mixture of beetroot extract and maltodextrin; 0.4% *w*/*w* betanin), pure betanin, and spray-dried beetroot juice, in W/O/W emulsions prepared using a straight-through microfluidic device. W/O single emulsions were previously prepared using betanin (0.1–1.0% *w*/*w*) and D-glucose (1% *w*/*w*) as the dispersed aqueous phase, while soybean oil and a tetraglycerin monolaurate condensed ricinoleic acid ester emulsifier (CR-310; 1% *w*/*w*) were used as the continuous oil phase. These emulsions were inserted into the microfluidic device at a controlled flux (5 to 100 L/m^2^ h) and broken into droplets within the outer aqueous phase (aqueous solution of Tween 20, 1–3% *w*/*w*). Increasing the flux from 5 to 20 L/m^2^ h did not affect the droplet size. However, the droplet size and polydispersity increased when the flux was higher than 100 L/m^2^ h. The W/O/W emulsion encapsulating pure betanin showed smaller droplets, higher stability, and droplet size distribution when compared to the emulsions containing betanin from other sources and the negative control (without pigment), probably due to the higher electrostatic repulsion observed between these droplets.

**Figure 5:**
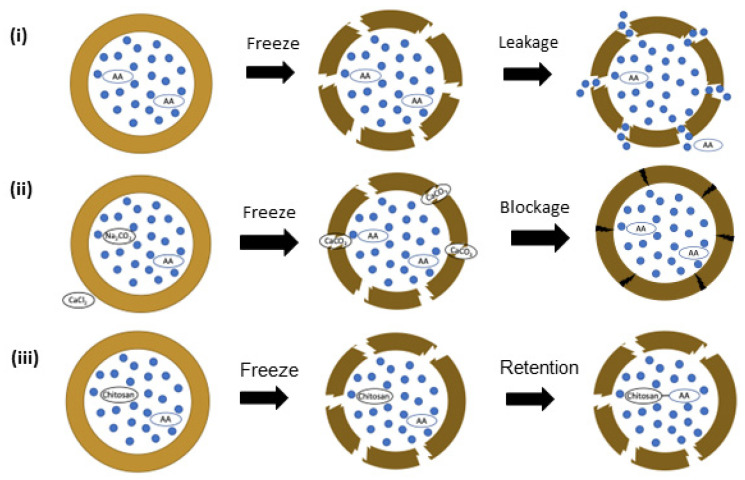
Schematic representation of the mechanisms involved in the release or retention of ascorbic acid (AA) in the core of solid lipid microcapsules (SLMs): (**i**) AA release through pores formed during lipid solidification, (**ii**) AA retention due to clogging of lipid pores at high salt concentrations (Na_2_CO_3_), and (**iii**) AA retention due to the chelating action of chitosan [11].

While W/O single emulsions are widely used as templates to prepare microgels, O/W/O and W/O/W double emulsions are generally used to fabricate microcapsules (Shah et al., 2008), including solid lipid microcapsules and core–shell microcapsules. Comunian et al. (2014) [11] used W/O/W emulsions generated in the capillary microfluidic devices as templates to design solid lipid microcapsules (SLMs) loaded with ascorbic acid. SLMs consist of a matrix made of solid lipids stabilized in an aqueous dispersion by surfactants or polymers [107]. Thus, melted palm fat was used as the middle phase. In contrast, the innermost aqueous phase and the continuous phase contained an ascorbic acid solution (3–20% *w*/*w*) with or without the presence of salt (Na_2_CO_3_) or/and chitosan (0.25% *w*/*w*) and only PVA (10% *w*/*v*), respectively. The W/O/W emulsion was collected in an ice bath to solidify the oil droplets to form a shell rapidly. The encapsulation efficiency of ascorbic acid increased from 73% to about 90% when salt and chitosan were added to the SLMs. Two different mechanisms to increase ascorbic acid’s encapsulation efficiency and stability were described: (1) the clogging of the pores generated during the lipid-solidification process at high salt concentrations and (2) the presence of chitosan inside the core, acting with a macromolecule-chelating agent (Figure 5) [11].

Although many studies point to core–shell microcapsules as candidates for drug delivery systems, most of the materials used for producing these systems by microfluidic techniques are synthetic polymers, including food-grade ones (e.g., PLGA) [75,97,106,108]. PLGA microcapsules encapsulating mesoporous silica nanoparticles (MSNs) were generated from the W/O/W emulsion via capillary microfluidic devices to obtain further, more specific control over drug release kinetics. MSNs can penetrate tissues through capillaries and be absorbed by cells, improving drug delivery to injury sites in the body. To generate W/O/W emulsions in microchannels, the innermost aqueous phase was composed of an MSN solution, while the PLGA solution (0.6 wt% in DCM) was injected into the microchannel as the middle oil phase. The PVA aqueous solution (outermost phase) flows through the square capillary from the opposite direction of the inner and middle phases. After double-emulsion generation, the PLGA microcapsules were solidified by the evaporation of DCM. The mean diameter of MSN-loaded PLGA microcapsules was 56 μm (CV = 4.91%). Furthermore, the release of a model dye from these microcapsules was sustained for 4 months without any observable burst release [97].

H. Chen et al. (2018) [106] used the same microfluidic approach to produce PLGA microcapsules loaded with 2-[[(4-phenoxyphenyl)sulfonyl]methyl]-thiirane (SB-3CT) for traumatic-brain-injury (TBI) pharmacological therapy. The PLGA-SB-3CT microcapsules with sizes ranging from 35 to 65 µm and an encapsulation efficiency of 99% presented an SB-3CT release at around 50 days. Then, PLGA-SB-3CT microcapsules injected in rats at the trauma site after TBI showed preliminary neuronal protection efficacy by accelerating behavioral recovery and reducing neuronal cell apoptosis in the CA2, hilus hippocampus, and cortical-injury region. In another study, core–shell microparticles were produced from W/O/W emulsion templates using gelatin-methacryloyl (GelMa) as the core and PLGA oil solution as the shell for synergistic and sustained drug delivery applications [108]. GelMa is a semisynthetic material obtained from the reaction of gelatin with methacrylic anhydride, which results in the modification of hydroxyl and lysine residues with methacrylate and methacrylamide side groups [109]. The GelMa core added with doxorubicin hydrochloride (DOX, hydrophilic drug) was photopolymerized under UV illumination downstream from the capillary microfluidic channel, while the PLGA shell added with camptothecine (CPT, hydrophobic drug) was solidified after DCM evaporation. Drug release from core–shell microcapsules initially occurred by diffusion of DOX from the GelMa core and delivery of DOX and CPT to the external environment through pores throughout the PLGA shell. With the degradation of the PLGA shell, the DOX and CPT drugs were also gradually released. Furthermore, liver cancer cells (HCT116 and HepG2) treated with GelMa-PLGA core–shell microcapsules loaded with DOX and CPT showed reduced viability (less than 20% for HCT116 cells and 10% for HepG2 cells), which confirmed the high therapeutic efficacy of these microcapsules in treating liver cancer cells [108].

Table S2 summarizes the technological approaches and properties of the delivery systems based on food-grade emulsions assembled by microfluidic techniques, including the microfluidic device type, emulsification process conditions, functional compound type, and delivery system characteristics (e.g., size, polydispersity, and encapsulation efficiency). Other recent studies have also pointed to microfluidics as an excellent tool for obtaining delivery systems based on food-grade emulsions. In general, these works aimed to evaluate droplet-formation process parameters using complex fluids and/or the development of new geometries or microfluidic devices without effective encapsulation of a functional compound. Considering their technological potential to act as delivery systems, Table S3 presents the most recent droplet-based microfluidics approaches to obtain emulsified systems, including the phase compositions, emulsification process conditions, and microfluidic device type.

## 4. Limitations and Perspectives of Scale-up in Droplet-Based Microfluidic Approaches

As aforementioned, many studies reported and supported the advantages of using droplet-based microfluidics to encapsulate lipophilic and hydrophilic functional compounds. However, the path from proof-of-concept in developing delivery systems via microfluidic technologies to their practical application on an industrial scale presents some problems that limit this transition. Although microfluidic techniques allow the manipulation of a small volume of fluids and the precise control of droplet generation into the microchannels, their production efficiency cannot satisfy industrial productivity. On a laboratory scale, about milliliters of material can be generated per hour; however, the industrial-scale demands many tons per year, leading scientists and researchers to seek solutions.

**Figure 6:**
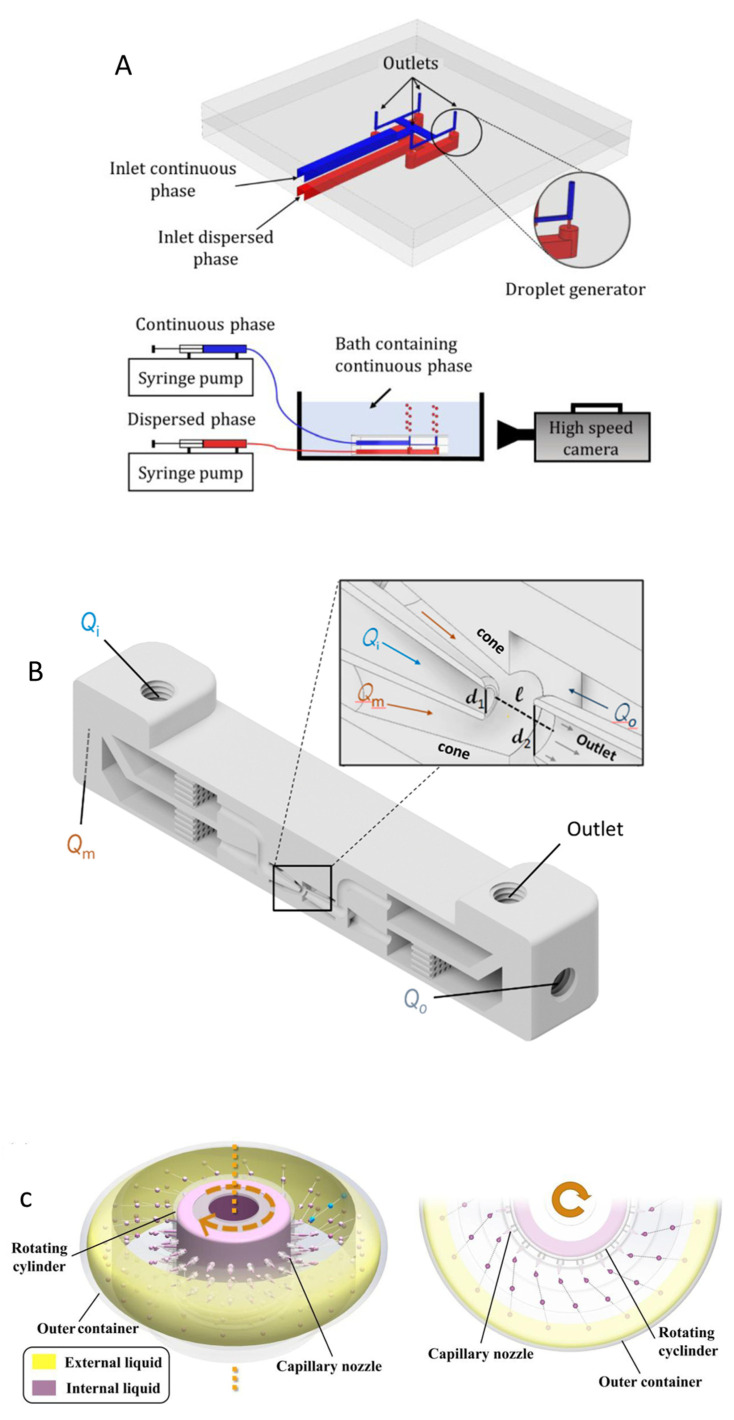
(**A**) Overall view of the 3D PMMA microdevice containing 4 nozzles, a droplet generator magnification, and the microfluidic setup schematic representation [114] (Gelin et al., 2020). (**B**) Lateral cut view on a 3D rendering of the parallelized device with one of the 4 double-emulsion droplet-makers and the respective distribution channels for 3 inlets and a collection channel. *Q_i_* shows the inlet of the inner phase, *Q_m_* of the middle phase, *Q_o_* of the continuous outer phase, and finally, the outlet for collecting the double emulsions is indicated [117]. (**C**) Multichannel rotating system [121].

As an alternative to industrial-scale, parallel microchannels in different configurations have been developed to solve the microdevice scalability issue. The most common scale-up devices are based on the parallelization (2D scale-up) and/or stacking (3D scale-up) of microchannels. This strategy of the parallelizing microchannel is called “numbering-up”, and recently, versatile platforms have been created to scale-up droplet formation [110,111]. In 2D scale-up, multiple channels with identical junctions are designed on a single chip for simultaneous droplet generation. These chips can also be stacked to form a parallel multichip system. Mulligan and Rothstein (2012) [112] developed a PDMS microfluidic device composed of six flow-focusing junctions operating in parallel to generate W/O single emulsions. The continuous phase, composed of mineral oil, was pumped at a flow rate (*Qc*) of 20 µL/min, while the dispersed aqueous phase, composed of surfactant solution and 5 mM cetylpyridinium chloride (CPyCL), was pumped at a varied flow rate (*Qd* = 2–20 µL/min). All microchannels were fed with only two inlets, one for each phase, and the effects of the flow rates on the droplet-generation process were analyzed. The emulsion droplets had sizes varying from 155 to 179 µm and a coefficient of variation (*CV*) of 5%, slightly higher than emulsions produced in only one channel junction (*CV*~2%). Probably, the fluid distribution affected the pressure inside the microchannels, disfavoring the formation of more uniform droplets [113]. In another study, Gelin et al. (2020) [114] designed a 3D PMMA microdevice with four parallel droplet generators to produce O/W single emulsions (Figure 6A). In this system, the flow rate of the continuous phase (aqueous solution of PVA 2 wt%) and the dispersed phase (mixture of hexane and Span 80 20 wt%) varied between 1 and 4 mL/h (*Qd* = *Qc*). At low flow rates, large droplets with high polydispersity were generated. By increasing the flow rates to 4 mL/h, smaller and monodisperse droplets could be produced in the parallelized microchannels. 

Some companies have also been exploring the development of innovative microfluidic devices through parallelization and stacking microchannels to turn them into commercially successful products. For example, Dolomite has developed Telos^®^ Chips (Table S1), glass microfluidic devices with seven parallel junctions to achieve higher throughput for various applications. This chip can be stacked with 10 more, resulting in 70-droplet generators [115]. In addition, Microfluidic ChipShop designs the Fluidic 912 (Table S1), a droplet-generation chip made of Topas^®^ COC (cyclic olefin copolymer) or polycarbonate (PC) that consists of eight parallel flow-focusing junctions. The company also has a smaller microchip with three-droplet generators (Fluidic 536), which could be combined into a three-dimensional model to increase droplet production [116]. 

The straight-through microfluidic devices and their variations, called step emulsification, have also been great alternatives in simultaneous droplet-generation processes [113]. Additionally, more complex configurations of parallel microfluidic devices have been designed to obtain multiple emulsions [117]. As mentioned, multiple emulsions, such as O/W/O and W/O/W emulsions, can be widely exploited in pharmaceutical and medical applications, including for multidrug delivery. Using 3D printing and acrylic monomer, Jans et al. (2019) [117] created a microfluidic device with four identical parallel structures for the high-throughput synthesis of W/O/W and O/W/O (diameter 500 µm) emulsions (Figure 6B). The 3D-printed microdevice, combining lithography patterning with capillary geometry, exhibits good resolution for generating small and monodisperse droplets.

Moreover, it has a low dead volume and high productivity (production rate of 12 L/h and 10^7^ monodisperse microgels per hour). Despite these advantages, challenges persist in manufacturing, cleaning, and maintaining these devices. For example, if a single channel becomes clogged, the entire device becomes inoperative until maintenance is performed. Consequently, developing high-throughput droplet-generating devices with independent maintenance and cleaning capabilities may offer exciting alternatives for future applications. 

A unique high-throughput droplet-generation microchip is desirable because scalability is related to droplet production, not just scaling microchannel size or chip quantity. Thus, the main issue in scaling-up droplet-based microchips is to produce monodisperse droplets in an easy operation and faster generation. This challenge can be overcome using a step-emulsion microfluidic with many parallelized microchannels that generate droplets simultaneously [118,119]. ]. One phase flows into one hundred identical microchannels, intersecting an upper reservoir channel containing another phase. Rutte et al. (2019) [118] developed a step-emulsion microdevice in which the droplet generation was induced by geometry and facilitated by the sudden expansion in channel height at the end of each channel. This microchip operates with flow rates between 5 and 10 mL/h; in contrast, a flow-focusing device operates at the magnitude of microliter per hour. Furthermore, the droplets presented sizes around 50 and 90 µm (CV 3%), determined by the microchannel dimensions. The droplets were gelled and used for tissue-engineering purposes; however, this microchip also has the potential to be used for generating previously unexplored food-grade droplets. 

Lastly, the parallelization of microchannels in nonspontaneous droplet-generation methods, called active, has been explored as a potential alternative to passive ones for developing food-grade emulsions on an industrial scale. In active methods, the droplet-formation process within the microchannels occurs under external forces, such as magnetic fields, electric fields, centrifuge forces, and acoustic waves [120,121,122]. Using a multichannel rotating system, J. Li et al. (2022) [121] produced alginate microgels (Figure 6C). In this system, the rotational cylinder is a core component that contains many glass capillary nozzles distributed like spikes on the periphery. The rotating cylinder serves as a reservoir for the dispersed phase and can rotate under the drive of a motor. Additionally, an outer container serves the rotor with the continuous phase. When the rotor is active during the centrifugation, the dispersed phase is thrown out to form pendant droplets through the capillary nozzle tips. The droplets detach from the tips when the centrifuge forces exceed the counteracting drawing force due to interfacial tension. These droplets then move until they fall into the continuous phase in the outer container [121]. The multichannel rotating system is an alternative for large-scale droplet production, since it can be adjusted to generate different emulsions with a desired dispersity and size at a production rate that can supply the needs of the industry.

## 5. Conclusions and Future Perspectives

Assembling delivery systems based on food-grade emulsions in microfluidic platforms requires extensive research for the effective implementation in food, pharmaceutical, medical, and cosmetics applications. Therefore, this review discussed the state-of-the-art droplet-based microfluidics for producing food-grade emulsions due to their importance as templates for developing more complex delivery systems and designing the properties of these systems depending on the entrapped compound type. Several microfluidic devices (e.g., planar, capillary, and straight-through) have been successfully employed to develop these food-grade systems, each with specific designs and characteristics. Microfluidic techniques also enabled, through the choice of ingredients and the emulsification process parameters, the precise modulation of the properties of the carrier systems to suit the type of encapsulated functional compound and its final application. 

Despite these advantages, some challenges associated with the economic aspects of the mass production, cleaning, and maintenance of the devices have limited the transition of microfluidic processes from the lab-scale to the industrial-scale. Addressing these economic aspects could make microfluidics more applicable to the food industry. However, achieving this goal necessitates research in scale-up, new materials, and process engineering. The main costs include those related to microfabrication and operation demands. Microfabrication costs encompass constructing and maintaining clean rooms, using oxygen (O_2_) plasma for device sealing, and using qualified techniques for fabrication. The most expensive components for operating a microfluidic device are syringe pumps and inverted microscopes. Furthermore, scaling up microfluidics is challenging due to the limited knowledge about the phenomenon. So, future studies focusing on developing high-throughput droplet-generating devices with independent maintenance and cleaning are needed, especially because complex fluids, such as biopolymers, are uneasy-flowing materials at high viscosities and prone to microchannel clogging. 

## Figures and Tables

**Table 1:** Advantages and limitations of different geometry droplet-based microfluidic devices.

Geometry	Advantages	Limitations
Planar	- Can be manufactured in different geometries (T- and Y-junction) - Easy to modulate and can be made with different materials (e.g., PDMS, PMMA, and other polymers) - Allow manufacturing more complex configuration chips	- Complex structures for industrial production - For multiple emulsions, it is a necessarily complex structure - Some based materials are not compatible with organic solvent - Rather sample microchannels structure
Capillary	- Good for multiple emulsions in one step - Commonly manufactured of glass, which has high transparency, high chemical resistance, inertness to most substances, and biocompatibility	- Complex structures for industrial production - Difficult reproducibility and manufacture - Low reproducibility - High cost - Highly time-consuming for fabrication
Straight-through	- Droplet generation independent of flow rate - Good monodispersity - Low energy consumption - Easy amplification of the device - Nondead volume - Reduced cost of chip design, processing, and operation	- Specific configuration

## Data Availability

Data is contained within the article or Supplementary Material.

## Supplementary Materials

The following supporting information can be downloaded at: https://www.mdpi.com/article/10.3390/foods12183385/s1, Table S1: Commercial microfluidic devices used to generate emulsion droplets; Table S2: Technological approaches and properties of the delivery systems based on food-grade emulsions assembled by microfluidic techniques; Table S3: Trends in droplet-based microfluidics approaches for fabricating delivery systems based on emulsions. References [115,116,123,124,125,126,127,128,129,130,131,132,133,134,135] are cited in Supplementary Materials.

## Abbreviations

PDMS	Polydimethylsiloxane
PMMA	Polymethyl methacrylate
O/W	Oil–water emulsion
O/W/O	Oil–water–oil emulsion
W/O	Water–oil emulsion
W/O/W	Water–oil–water emulsion
DRIE	Deep-reactive-ion etching
MC module	Microfluidic module
Ca	Capillary number
We	Weber number
U	Characteristic velocity of the fluid
µ	Dynamic viscosity
*l*	Characteristic dimension of the channel
*γ*	Interfacial tension
PGPR	Polyglycerol polyricinoleate
BSA	Bovine serum albumin
SDS	Sodium dodecyl sulfate
MO-7S	Decaglycerol monooleate
ML-750	Decaglycerol monolaurate
ML	Modified lecithin
Na-Cs	Sodium caseinate
MCT	Medium-chain triglycerides
LCT	Long-chain triglycerides
EDTA-Ca	Ethylenediaminetetraacetic acid calcium disodium salt hydrate
PEI	Polyethyleneimine
PLGA	Poly-lactic-co-glycolic acid
PVA	Poly (vinyl alcohol)
PEG	Polyethylene glycol
SHM	Staggered herringbone mixers
DSS	Dextran sulfate sodium
CR-310	Condensed ricinoleic acid ester
SLMs	Solid lipid microcapsules
MSNs	Mesoporous silica nanoparticles
SB-3CT	2-[[(4-phenoxyphenyl)sulfonyl]methyl]-thiirane
TBI	Traumatic brain injury
DOX	Doxorubicin hydrochloride
CPT	Camptothecin
GelMa	Gelatin-methacryloyl

## References

[1] Whitesides G.M. (2006). The Origins and the Future of Microfluidics. Nature.

[2] Teh S.-Y., Lin R., Hung L.-H., Lee A.P. (2008). Droplet Microfluidics. Lab Chip.

[3] Gale B.K., Jafek A.R., Lambert C.J., Goenner B.L., Moghimifam H., Nze U.C., Kamarapu S.K. (2018). A Review of Current Methods in Microfluidic Device Fabrication and Future Commercialization Prospects. Inventions.

[4] Yager P., Edwards T., Fu E., Helton K., Nelson K., Tam M.R., Weigl B.H. (2006). Microfluidic Diagnostic Technologies for Global Public Health. Nature.

[5] Dittrich P.S., Tachikawa K., Manz A. (2006). Micro Total Analysis Systems. Latest Advancements and Trends. Anal. Chem..

[6] Vit F.F., Nunes R., Wu Y.T., Prado Soares M.C., Godoi N., Fujiwara E., Carvalho H.F., Gaziola de la Torre L. (2021). A Modular, Reversible Sealing, and Reusable Microfluidic Device for Drug Screening. Anal. Chim. Acta.

[7] Damiati S., Kompella U.B., Damiati S.A., Kodzius R. (2018). Microfluidic Devices for Drug Delivery Systems and Drug Screening-a-Chip; Organ-on-a-Chip; Human-on-a-Chip. Genes.

[8] Lonchamps P.L., He Y., Wang K., Lu X. (2022). Detection of Pathogens in Foods Using Microfluidic “Lab-on-Chip”: A Mini Review. J. Agric. Food Res..

[9] Seemann R., Brinkmann M., Pfohl T., Herminghaus S. (2012). Droplet Based Microfluidics. Rep. Prog. Phys..

[10] Yamamoto F., Cunha R.L. (2007). Acid Gelation of Gellan: Effect of Final PH and Heat Treatment Conditions. Carbohydr. Polym..

[11] Comunian T.A., Abbaspourrad A., Favaro-Trindade C.S., Weitz D.A. (2014). Fabrication of Solid Lipid Microcapsules Containing Ascorbic Acid Using a Microfluidic Technique. Food Chem..

[12] McClements D.J., Decker E.A., Weiss J. (2007). Emulsion-Based Delivery Systems for Lipophilic Bioactive Components. J. Food Sci..

[13] Tan C., McClements D.J. (2021). Application of Advanced Emulsion Technology in the Food Industry: A Review and Critical Evaluation. Foods.

[14] Costa A.L.R., Gomes A., Ushikubo F.Y., Cunha R.L. (2017). Gellan Microgels Produced in Planar Microfluidic Devices. J. Food Eng..

[15] Santana R.C., Perrechil F.A., Cunha R.L. (2013). High- and Low-Energy Emulsifications for Food Applications: A Focus on Process Parameters. Food Eng. Rev..

[16] Khalid N., Kobayashi I., Neves M.A., Uemura K., Nakajima M., Nabetani H. (2017). Encapsulation of β-Sitosterol plus γ-Oryzanol in O/W Emulsions: Formulation Characteristics and Stability Evaluation with Microchannel Emulsification. Food Bioprod. Process..

[17] Santos T.P., Michelon M., Carvalho M.S., Cunha R.L. (2021). Formation and Stability of Oil-in-Water Emulsions Based on Components of Bioprocesses: A Microfluidic Analysis. Colloids Surf. A Physicochem. Eng. Asp..

[18] Yang L., Chu J.S., Fix J.A. (2002). Colon-Specific Drug Delivery: New Approaches and in Vitro/in Vivo Evaluation. Int. J. Pharm..

[19] Forigua A., Kirsch R.L., Willerth S.M., Elvira K.S. (2021). Recent Advances in the Design of Microfluidic Technologies for the Manufacture of Drug Releasing Particles. J. Control. Release.

[20] Shah R.K., Shum H.C., Rowat A.C., Lee D., Agresti J.J., Utada A.S., Chu L.Y., Kim J.W., Fernandez-Nieves A., Martinez C.J. (2008). Designer Emulsions Using Microfluidics. Mater. Today.

[21] Shao C., Chi J., Shang L., Fan Q., Ye F. (2022). Droplet Microfluidics-Based Biomedical Microcarriers. Acta Biomater..

[22] Zuidam N.J., Velikov K.P. (2018). Choosing the Right Delivery Systems for Functional Ingredients in Foods: An Industrial Perspective. Curr. Opin. Food Sci..

[23] Hinman S.S., Wang Y., Kim R., Allbritton N.L. (2020). In Vitro Generation of Self-Renewing Human Intestinal Epithelia over Planar and Shaped Collagen Hydrogels. Nat. Protoc..

[24] Ling K., Wu H., Neish A.S., Champion J.A. (2019). Alginate/Chitosan Microparticles for Gastric Passage and Intestinal Release of Therapeutic Protein Nanoparticles. J. Control. Release.

[25] Santos T.P., Costa A.L.R., Michelon M., Costa L.P., Cunha R.L. (2020). Development of a Microfluidic Route for the Formation of Gellan-Based Microgels Incorporating Jabuticaba (*Myrciaria cauliflora*) Extract. J. Food Eng..

[26] Feng Y., Lee Y. (2019). Microfluidic Assembly of Food-Grade Delivery Systems: Toward Functional Delivery Structure Design. Trends Food Sci. Technol..

[27] Kim J.H., Na J., Bak D.H., Lee B.C., Lee E., Choi M.J., Ryu C.H., Lee S., Mun S.K., Park B.C. (2019). Development of Finasteride Polymer Microspheres for Systemic Application in Androgenic Alopecia. Int. J. Mol. Med..

[28] Kim J.H., Ryu C.H., Chon C.H., Kim S., Lee S., Maharjan R., Kim N.A., Jeong S.H. (2021). Three Months Extended-Release Microspheres Prepared by Multi-Microchannel Microfluidics in Beagle Dog Models. Int. J. Pharm..

[29] Zhang K., Ren Y., Jiang T., Jiang H. (2022). Thermal Field-Actuated Multifunctional Double-Emulsion Droplet Carriers: On-Demand Migration, Core Release and Released Particle Focusing. Chem. Eng. J..

[30] Zhu P., Wang L. (2016). Passive and Active Droplet Generation with Microfluidics: A Review. Lab Chip.

[31] Shi M., Shi Y.L., Li X.M., Yang R., Cai Z.Y., Li Q.S., Ma S.C., Ye J.H., Lu J.L., Liang Y.R. (2018). Food-Grade Encapsulation Systems for (−)-Epigallocatechin Gallate. Molecules.

[32] Samal J., Segura T. (2021). Injectable Biomaterial Shuttles for Cell Therapy in Stroke. Brain Res. Bull..

[33] Liu Z.M., Yang Y., Du Y., Pang Y. (2017). Advances in Droplet-Based Microfluidic Technology and Its Applications. Chin. J. Anal. Chem..

[34] Logesh D., Vallikkadan M.S., Leena M.M., Moses J.A., Anandharamakrishnan C. (2021). Advances in Microfluidic Systems for the Delivery of Nutraceutical Ingredients. Trends Food Sci. Technol..

[35] Schroen K., Berton-Carabin C., Renard D., Marquis M., Boire A., Cochereau R., Amine C., Marze S. (2021). Droplet Microfluidics for Food and Nutrition Applications. Micromachines.

[36] Baroud C.N., Gallaire F., Dangla R. (2010). Dynamics of Microfluidic Droplets. Lab Chip.

[37] Atencia J., Beebe D.J. (2004). Controlled Microfluidic Interfaces. Nature.

[38] Costa A.L.R., Gomes A., Cunha R.L. (2017). Studies of Droplets Formation Regime and Actual Flow Rate of Liquid-Liquid Flows in Flow-Focusing Microfluidic Devices. Exp. Therm. Fluid Sci..

[39] Kobayashi I., Nakajima M., Chun K., Kikuchi Y., Fujita H. (2002). Silicon Array of Elongated Through-Holes for Monodisperse Emulsion Droplets. AIChE J..

[40] Sugiura S., Nakajima M., Iwamoto S., Seki M. (2001). Interfacial Tension Driven Monodispersed Droplet Formation from Microfabricated Channel Array. Langmuir.

[41] Ushikubo F.Y., Birribilli F.S., Oliveira D.R.B., Cunha R.L. (2014). Y- and T-Junction Microfluidic Devices: Effect of Fluids and Interface Properties and Operating Conditions. Microfluid. Nanofluid..

[42] Shui L., Van Den Berg A., Eijkel J.C.T. (2009). Interfacial Tension Controlled W/O and O/W 2-Phase Flows in Microchannel. Lab Chip.

[43] Khalid N., Shu G., Kobayashi I., Nakajima M., Barrow C.J. (2017). Formulation and Characterization of Monodisperse O/W Emulsions Encapsulating Astaxanthin Extracts Using Microchannel Emulsification: Insights of Formulation and Stability Evaluation. Colloids Surfaces B Biointerfaces.

[44] Pagano A.P.E., Khalid N., Kobayashi I., Nakajima M., Neves M.A., Bastos E.L. (2018). Microencapsulation of Betanin in Monodisperse W/O/W Emulsions. Food Res. Int..

[45] Khalid N., Kobayashi I., Neves M.A., Uemura K., Nakajima M., Nabetani H. (2016). Microchannel Emulsification Study on Formulation and Stability Characterization of Monodisperse Oil-in-Water Emulsions Encapsulating Quercetin. Food Chem..

[46] Dias Meirelles A.A., Rodrigues Costa A.L., Michelon M., Viganó J., Carvalho M.S., Cunha R.L. (2022). Microfluidic Approach to Produce Emulsion-Filled Alginate Microgels. J. Food Eng..

[47] Duncanson W.J., Lin T., Abate A.R., Seiffert S., Shah R.K., Weitz D.A. (2012). Microfluidic Synthesis of Advanced Microparticles for Encapsulation and Controlled Release. Lab Chip.

[48] McDonald J.C., Duffy D.C., Anderson J.R., Chiu D.T., Wu H., Schueller O.J.A., Whitesides G.M. (2000). Fabrication of Microfluidic Systems in Poly(Dimethylsiloxane). Electrophoresis.

[49] McDonald J.C., Whitesides G.M. (2002). Poly(Dimethylsiloxane) as a Material for Fabricating Microfluidic Devices. Acc. Chem. Res..

[50] Duffy D.C., McDonald J.C., Schueller O.J.A., Whitesides G.M. (1998). Rapid Prototyping of Microfluidic Systems in Poly(Dimethylsiloxane). Anal. Chem..

[51] Abate A.R., Poitzsch A., Hwang Y., Lee J., Czerwinska J., Weitz D.A. (2009). Impact of Inlet Channel Geometry on Microfluidic Drop Formation. Phys. Rev. E–Stat. Nonlinear Soft Matter Phys..

[52] Roberts C.C., Rao R.R., Loewenberg M., Brooks C.F., Galambos P., Grillet A.M., Nemer M.B. (2012). Comparison of Monodisperse Droplet Generation in Flow-Focusing Devices with Hydrophilic and Hydrophobic Surfaces. Lab Chip.

[53] Vladisavljević G.T., Kobayashi I., Nakajima M. (2012). Production of Uniform Droplets Using Membrane, Microchannel and Microfluidic Emulsification Devices. Microfluid. Nanofluid..

[54] Zhao C.X. (2013). Multiphase Flow Microfluidics for the Production of Single or Multiple Emulsions for Drug Delivery. Adv. Drug Deliv. Rev..

[55] Tarchichi N., Chollet F., Manceau J.F. (2013). New Regime of Droplet Generation in a T-Shape Microfluidic Junction. Microfluid. Nanofluid..

[56] Wu P., Luo Z., Liu Z., Li Z., Chen C., Feng L., He L. (2015). Drag-Induced Breakup Mechanism for Droplet Generation in Dripping within Flow Focusing Microfluidics. Chin. J. Chem. Eng..

[57] Collins D.J., Neild A., deMello A., Liu A.Q., Ai Y. (2015). The Poisson Distribution and beyond: Methods for Microfluidic Droplet Production and Single Cell Encapsulation. Lab Chip.

[58] Costa A.L.R., Gomes A., de Andrade C.C.P., Cunha R.L. (2017). Emulsifier Functionality and Process Engineering: Progress and Challenges. Food Hydrocoll..

[59] Fu T., Wu Y., Ma Y., Li H.Z. (2012). Droplet Formation and Breakup Dynamics in Microfluidic Flow-Focusing Devices: From Dripping to Jetting. Chem. Eng. Sci..

[60] Battat S., Weitz D.A., Whitesides G.M. (2022). Nonlinear Phenomena in Microfluidics. Chem. Rev..

[61] Fuciños C., Rodríguez-Sanz A., García-Caamaño E., Gerbino E., Torrado A., Gómez-Zavaglia A., Rúa M.L. (2023). Microfluidics Potential for Developing Food-Grade Microstructures through Emulsification Processes and Their Application. Food Res. Int..

[62] Christopher G.F., Anna S.L. (2007). Microfluidic Methods for Generating Continuous Droplet Streams. J. Phys. D. Appl. Phys..

[63] Garti N., McClements D.J. (2012). Encapsulation Technologies and Delivery Systems for Food Ingredients and Nutraceuticals.

[64] Qian C., Decker E.A., Xiao H., McClements D.J. (2012). Inhibition of β-Carotene Degradation in Oil-in-Water Nanoemulsions: Influence of Oil-Soluble and Water-Soluble Antioxidants. Food Chem..

[65] Dammak I., de Carvalho R.A., Trindade C.S.F., Lourenço R.V., do Amaral Sobral P.J. (2017). Properties of Active Gelatin Films Incorporated with Rutin-Loaded Nanoemulsions. Int. J. Biol. Macromol..

[66] Murillo A.G., Aguilar D., Norris G.H., DiMarco D.M., Missimer A., Hu S., Smyth J.A., Gannon S., Blesso C.N., Luo Y. (2016). Compared with Powdered Lutein, a Lutein Nanoemulsion Increases Plasma and Liver Lutein, Protects against Hepatic Steatosis, and Affects Lipoprotein Metabolism in Guinea Pigs. J. Nutr..

[67] Ozturk B., Argin S., Ozilgen M., McClements D.J. (2015). Formation and Stabilization of Nanoemulsion-Based Vitamin E Delivery Systems Using Natural Biopolymers: Whey Protein Isolate and Gum Arabic. Food Chem..

[68] Costa A.L.R., Gomes A., de F. Furtado G., Tibolla H., Menegalli F.C., Cunha R.L. (2020). Modulating in Vitro Digestibility of Pickering Emulsions Stabilized by Food-Grade Polysaccharides Particles. Carbohydr. Polym..

[69] Gomes A., Costa A.L.R., Cardoso D.D., Náthia-Neves G., Meireles M.A.A., Cunha R.L. (2021). Interactions of β-Carotene with WPI/Tween 80 Mixture and Oil Phase: Effect on the Behavior of O/W Emulsions during in Vitro Digestion. Food Chem..

[70] Jafari S.M., Assadpoor E., He Y., Bhandari B. (2008). Re-Coalescence of Emulsion Droplets during High-Energy Emulsification. Food Hydrocoll..

[71] Leser M.E., Sagalowicz L., Michel M., Watzke H.J. (2006). Self-Assembly of Polar Food Lipids. Adv. Colloid Interface Sci..

[72] Ma Z., Zhao Y., Khalid N., Shu G., Neves M.A., Kobayashi I., Nakajima M. (2020). Comparative Study of Oil-in-Water Emulsions Encapsulating Fucoxanthin Formulated by Microchannel Emulsification and High-Pressure Homogenization. Food Hydrocoll..

[73] Gomes A., Costa A.L.R., Cunha R.L. (2018). Impact of Oil Type and WPI/Tween 80 Ratio at the Oil-Water Interface: Adsorption, Interfacial Rheology and Emulsion Features. Colloids Surf. B Biointerfaces.

[74] Gomes A., Furtado G.D.F., Cunha R.L. (2019). Bioaccessibility of Lipophilic Compounds Vehiculated in Emulsions: Choice of Lipids and Emulsifiers. J. Agric. Food Chem..

[75] Chen Z., Song S., Ma J., Da Ling S., Wang Y.D., Kong T.T., Xu J.H. (2022). Fabrication of Magnetic Core/Shell Hydrogels via Microfluidics for Controlled Drug Delivery. Chem. Eng. Sci..

[76] Mou C.L., Deng Q.Z., Hu J.X., Wang L.Y., Deng H.B., Xiao G., Zhan Y. (2020). Controllable Preparation of Monodisperse Alginate Microcapsules with Oil Cores. J. Colloid Interface Sci..

[77] Sek L., Porter C.J.H., Kaukonen A.M., Charman W.N. (2010). Evaluation of the In-Vitro Digestion Profiles of Long and Medium Chain Glycerides and the Phase Behaviour of Their Lipolytic Products. J. Pharm. Pharmacol..

[78] Santos T.P., Cunha R.L. (2019). In Vitro Digestibility of Gellan Gels Loaded with Jabuticaba Extract: Effect of Matrix-Bioactive Interaction. Food Res. Int..

[79] De Moura S.C.S.R., Berling C.L., Garcia A.O., Queiroz M.B., Alvim I.D., Hubinger M.D. (2019). Release of Anthocyanins from the Hibiscus Extract Encapsulated by Ionic Gelation and Application of Microparticles in Jelly Candy. Food Res. Int..

[80] Soukoulis C., Tsevdou M., Andre C.M., Cambier S., Yonekura L., Taoukis P.S., Hoffmann L. (2017). Modulation of Chemical Stability and In Vitro Bioaccessibility of Beta-Carotene Loaded in Kappa-Carrageenan Oil-in-Gel Emulsions. Food Chem..

[81] Zhang J., Xu W., Xu F., Lu W., Hu L., Zhou J., Zhang C., Jiang Z. (2021). Microfluidic Droplet Formation in Co-Flow Devices Fabricated by Micro 3D Printing. J. Food Eng..

[82] Ahmed E.M. (2015). Hydrogel: Preparation, Characterization, and Applications: A Review. J. Adv. Res..

[83] Lee J.H. (2018). Injectable Hydrogels Delivering Therapeutic Agents for Disease Treatment and Tissue Engineering. Biomater. Res..

[84] Pan X., Mercadé-Prieto R., York D., Preece J.A., Zhang Z. (2013). Structure and Mechanical Properties of Consumer-Friendly PMMA Microcapsules. Ind. Eng. Chem. Res..

[85] Vu H.T.H., Streck S., Hook S.M., McDowell A. (2019). Utilization of Microfluidics for the Preparation of Polymeric Nanoparticles for the Antioxidant Rutin: A Comparison with Bulk Production. Pharm. Nanotechnol..

[86] Anderson J.M., Shive M.S. (1997). Biodegradation and Biocompatibility of PLA and PLGA Microspheres. Adv. Drug Deliv. Rev..

[87] Rezvantalab S., Keshavarz Moraveji M. (2019). Microfluidic Assisted Synthesis of PLGA Drug Delivery Systems. RSC Adv..

[88] Kim S., Wang H., Yan L., Zhang X., Cheng Y. (2020). Continuous Preparation of Itraconazole Nanoparticles Using Droplet-Based Microreactor. Chem. Eng. J..

[89] Lorenz T., Bojko S., Bunjes H., Dietzel A. (2018). An Inert 3D Emulsification Device for Individual Precipitation and Concentration of Amorphous Drug Nanoparticles. Lab Chip.

[90] Liu Y., Huang L., Liu F. (2010). Paclitaxel Nanocrystals for Overcoming Multidrug Resistance in Cancer. Mol. Pharm..

[91] Petros R.A., Desimone J.M. (2010). Strategies in the Design of Nanoparticles for Therapeutic Applications. Nat. Rev. Drug Discov..

[92] Williams M.S., Longmuir K.J., Yager P. (2008). A Practical Guide to the Staggered Herringbone Mixer. Lab Chip.

[93] Michelon M., Huang Y., de la Torre L.G., Weitz D.A., Cunha R.L. (2019). Single-Step Microfluidic Production of W/O/W Double Emulsions as Templates for β-Carotene-Loaded Giant Liposomes Formation. Chem. Eng. J..

[94] Favaro-Trindade C.S., Santana A.S., Monterrey-Quintero E.S., Trindade M.A., Netto F.M. (2010). The Use of Spray Drying Technology to Reduce Bitter Taste of Casein Hydrolysate. Food Hydrocoll..

[95] Kuang S.S., Oliveira J.C., Crean A.M. (2010). Microencapsulation as a Tool for Incorporating Bioactive Ingredients into Food. Crit. Rev. Food Sci. Nutr..

[96] Yu W., Liu X., Zhao Y., Chen Y. (2019). Droplet Generation Hydrodynamics in the Microfluidic Cross-Junction with Different Junction Angles. Chem. Eng. Sci..

[97] Zhou C., Zhu P., Han X., Shi R., Tian Y., Wang L. (2021). Microfluidic Generation of ATPS Droplets by Transient Double Emulsion Technique. Lab Chip.

[98] Tumarkin E., Kumacheva E. (2009). Microfluidic Generation of Microgels from Synthetic and Natural Polymers. Chem. Soc. Rev..

[99] Milivojevic M., Pajic-Lijakovic I., Bugarski B., Nayak A.K., Hasnain M.S. (2019). Gellan Gum in Drug Delivery Applications. Nat. Polysaccharides Drug Deliv. Biomed. Appl..

[100] Ogończyk D., Siek M., Garstecki P. (2011). Microfluidic Formulation of Pectin Microbeads for Encapsulation and Controlled Release of Nanoparticles. Biomicrofluidics.

[101] Montalbano G., Toumpaniari S., Popov A., Duan P., Chen J., Dalgarno K., Scott W.E., Ferreira A.M. (2018). Synthesis of Bioinspired Collagen/Alginate/Fibrin Based Hydrogels for Soft Tissue Engineering. Mater. Sci. Eng. C.

[102] Yu L., Sun Q., Hui Y., Seth A., Petrovsky N., Zhao C.X. (2019). Microfluidic Formation of Core-Shell Alginate Microparticles for Protein Encapsulation and Controlled Release. J. Colloid Interface Sci..

[103] Estrada L.H., Chu S., Champion J.A. (2014). Protein Nanoparticles for Intracellular Delivery of Therapeutic Enzymes. J. Pharm. Sci..

[104] Herrera Estrada L., Wu H., Ling K., Zhang G., Sumagin R., Parkos C.A., Jones R.M., Champion J.A., Neish A.S. (2017). Bioengineering Bacterially Derived Immunomodulants: A Therapeutic Approach to Inflammatory Bowel Disease. ACS Nano.

[105] Noh J., Kim J., Kim J.S., Chung Y.S., Chang S.T., Park J. (2018). Microencapsulation by Pectin for Multi-Components Carriers Bearing Both Hydrophobic and Hydrophilic Active Agents. Carbohydr. Polym..

[106] Chen H., Jia F., Zhu C., Xu J., Hua X., Xi Z., Shen L., Zhao S., Cen L. (2018). Controllable Preparation of SB-3CT Loaded PLGA Microcapsules for Traumatic-Brain-Injury Pharmaco-Therapy. Chem. Eng. J..

[107] Dingler A., Gohla S. (2008). Production of Solid Lipid Nanoparticles (SLN): Scaling up Feasibilities. J. Microencapsul..

[108] Li Y., Yan D., Fu F., Liu Y., Zhang B., Wang J., Shang L., Gu Z., Zhao Y. (2017). Composite Core-Shell Microparticles from Microfluidics for Synergistic Drug Delivery. Sci. China Mater..

[109] Yue K., Trujillo-de Santiago G., Alvarez M.M., Tamayol A., Annabi N., Khademhosseini A. (2015). Synthesis, Properties, and Biomedical Applications of Gelatin Methacryloyl (GelMA) Hydrogels. Biomaterials.

[110] Lin L., Quoc Pho H., Zong L., Li S., Pourali N., Rebrov E., Nghiep Tran N., Ostrikov K., Hessel V. (2021). Microfluidic Plasmas: Novel Technique for Chemistry and Chemical Engineering. Chem. Eng. J..

[111] Asadi-Saghandi H., Karimi-Sabet J., Ghorbanian S., Moosavian S.M.A. (2022). Dimensionless Analysis on Liquid–Liquid Two-Phase Flow Patterns in a Numbered-up Microfluidic Device. Chem. Eng. J..

[112] Mulligan M.K., Rothstein J.P. (2012). Scale-up and Control of Droplet Production in Coupled Microfluidic Flow-Focusing Geometries. Microfluid. Nanofluidics.

[113] Liu Z., Duan C., Jiang S., Zhu C., Ma Y., Fu T. (2020). Microfluidic Step Emulsification Techniques Based on Spontaneous Transformation Mechanism: A Review. J. Ind. Eng. Chem..

[114] Gelin P., Bihi I., Ziemecka I., Thienpont B., Christiaens J., Hellemans K., Maes D., De Malsche W. (2020). Microfluidic Device for High-Throughput Production of Monodisperse Droplets. ACS Appl. Mater. Interfaces.

[115] Dolomite Microfluidics 2022 Dolomite Microfluidics. https://www.dolomite-microfluidics.com/.

[116] Microfluidic ChipShop 2022 Microfluidic ChipShop. https://www.microfluidic-chipshop.com/.

[117] Jans A., Lölsberg J., Omidinia-Anarkoli A., Viermann R., Möller M., De Laporte L., Wessling M., Kuehne A.J.C. (2019). High-Throughput Production of Micrometer Sized Double Emulsions and Microgel Capsules in Parallelized 3D Printed Microfluidic Devices. Polymers.

[118] de Rutte J.M., Koh J., Di Carlo D. (2019). Scalable High-Throughput Production of Modular Microgels for In Situ Assembly of Microporous Tissue Scaffolds. Adv. Funct. Mater..

[119] Lee S., De Rutte J., Dimatteo R., Koo D., Di Carlo D. (2022). Scalable Fabrication and Use of 3D Structured Microparticles Spatially Functionalized with Biomolecules. ACS Nano.

[120] Hajji I., Serra M., Geremie L., Ferrante I., Renault R., Viovy J.L., Descroix S., Ferraro D. (2020). Droplet Microfluidic Platform for Fast and Continuous-Flow RT-QPCR Analysis Devoted to Cancer Diagnosis Application. Sens. Actuators B Chem..

[121] Li J., Wang Y., Cai L., Shang L., Zhao Y. (2022). High-Throughput Generation of Microgels in Centrifugal Multi-Channel Rotating System. Chem. Eng. J..

[122] Li Z., Paulson A.T., Gill T.A. (2015). Encapsulation of Bioactive Salmon Protein Hydrolysates with Chitosan-Coated Liposomes. J. Funct. Foods.

[123] Zhang J., Zhang R., Zhang Y., Pan Y., Shum H.C., Jiang Z. (2021). Alginate-Gelatin Emulsion Droplets for Encapsulation of Vitamin A by 3D Printed Microfluidics. Particuology.

[124] Zhou J., Zhai Y., Xu J., Zhou T., Cen L. (2021). Microfluidic Preparation of PLGA Composite Microspheres with Mesoporous Silica Nanoparticles for Finely Manipulated Drug Release. Int. J. Pharm..

[125] Manickam S., Sivakumar K., Pang C.H. (2020). Investigations on the Generation of Oil-in-Water (O/W) Nanoemulsions through the Combination of Ultrasound and Microchannel. Ultrason. Sonochem..

[126] Nehme R., Blel W., Montillet A., Bellettre J., Marchal L. (2021). Production of Oil in Water Emulsions in Microchannels at High Throughput: Evaluation of Emulsions in View of Cosmetic, Nutraceutical or Pharmaceutical Applications. Chem. Eng. Process.-Process Intensif..

[127] Taarji N., Vodo S., Bouhoute M., Khalid N., Hafidi A., Kobayashi I., Neves M.A., Isoda H., Nakajima M. (2020). Preparation of Monodisperse O/W Emulsions Using a Crude Surface-Active Extract from Argan by-Products in Microchannel Emulsification. Colloids Surfaces A Physicochem. Eng. Asp..

[128] Kim C.M., Choi H.J., Park E.J., Kim G.M. (2020). Repeated Geometrical T-Junction Breakup Microfluidic Filter Device by Injection of Premixed Emulsion for Microdroplet Production. J. Ind. Eng. Chem..

[129] Jurinjak Tušek A., Jurina T., Čulo I., Valinger D., Gajdoš Kljusurić J., Benković M. (2022). Application of NIRs Coupled with PLS and ANN Modelling to Predict Average Droplet Size in Oil-in-Water Emulsions Prepared with Different Microfluidic Devices. Spectrochim. Acta Part A Mol. Biomol. Spectrosc..

[130] Lacroix A., Hayert M., Bosc V., Menut P. (2022). Batch versus Microfluidic Emulsification Processes to Produce Whey Protein Microgel Beads from Thermal or Acidic Gelation. J. Food Eng..

[131] Keller S., Dekkers R., Hu G.X., Tollemeto M., Morosini M., Keskin A., Wilson D.A. (2021). A Simple Microfluidic Tool to Design Anisotropic Microgels. React. Funct. Polym..

[132] Leister N., Vladisavljević G.T., Karbstein H.P. (2022). Novel Glass Capillary Microfluidic Devices for the Flexible and Simple Production of Multi-Cored Double Emulsions. J. Colloid Interface Sci..

[133] Sattari A., Hanafizadeh P., Keshtiban M.M. (2021). Microfluidic Preparation of Double Emulsions Using a High Aspect Ratio Double Co-Flow Device. Colloids Surfaces A Physicochem. Eng. Asp..

[134] Bandulasena M.V., Vladisavljević G.T., Benyahia B. (2019). Versatile Reconfigurable Glass Capillary Microfluidic Devices with Lego^®^ Inspired Blocks for Drop Generation and Micromixing. J. Colloid Interface Sci..

[135] Cai Q.W., Ju X.J., Chen C., Faraj Y., Jia Z.H., Hu J.Q., Xie R., Wang W., Liu Z., Chu L.Y. (2019). Fabrication and Flow Characteristics of Monodisperse Bullet-Shaped Microparticles with Controllable Structures. Chem. Eng. J..

