# Supplementary material for: Droplet-Based Microfluidics as a Platform to Design Food-Grade Delivery Systems Based on the Entrapped Compound Type
Source: Foods. 2023 Sep 9;12(18):3385. doi: 10.3390/foods12183385 (PMC10527709; doi:10.3390/foods12183385)
Supplement: Supplementary file 1 [file foods-12-03385-s001.zip › foods-2594923-supplementary.pdf]

Table S1. Commercial microfluidic devices used to generate emulsion droplets.

Company	Brand	Material	Characteristics	Applications	References
Dolomite	Telos ® 2 regent<br>Telos ® 1 reagent<br>3D flow focusing SC	Glass microfluidic device	-The device consists of 7 parallel junctions that can be combined with others 10.<br>-Hydrophilic, hydrophobic, and fluorophilic coating options.<br>-Available at 100 or 50 µm.	Designed for emulsion generation, foam generation, micro-particle synthesis and high throughput experimentation (for example, analysis of cells).	[1]
	Telos ® micromixer Chip		-The device consists of 7 independent micromixer channels on the chip (with 5 mixing stages each).<br>-Hydrophilic and hydrophobic coating options.<br>-Available at 30 or 50 µm.	Designed to create nano and micro particles and emulsions.	
	µEncapsulator 2 Reagent Droplet Chip		-Chip size 11.25mm x 15mm x 2mm.<br>-Hydrophilic and fluorophilic coating surface options.<br>-Junction of 100 µm scale.	Designed for rapid generation of double emulsion droplets (an aqueous core, surrounded by an oil shell)	
	µEncapsulator Sample Reservoir Chip		-Smooth channel with 100 µL storage volume.<br>-Easy to clean and clear device.<br>-Hydrophilic coating surface.	Designed for single emulsion droplet generation.	
	Large Droplet Junction Chip	Glass and quartz device	-Chip offers both a T- and X-junction.<br>-Simple and easy to use.<br>-High droplet production (12,000 per second).<br>-Hydrophilic, hydrophilic and, fluorophilic coating options.	Designed for droplet emulsion generation.	
	Small Droplet Junction Chip		-Chip offers a flow focusing junction geometry with 14 x 17 µm cross-section at the junction.	Designed for generating small droplets in the size range of 5-30 µm,	

			-High droplet production (12,000 per second).<br>-Hydrophilic, hydrophilic and, fluorophilic coating options.	improving control over the targeting and release of active compounds.	
	T-Junction Chip		-Extremely smooth channel surface.<br>- Excellent chemical compatibility.<br>-Hydrophilic, hydrophilic and, fluorophilic coating options.	Designed for a range of applications, including mixing fluids, microreactions and droplet formation.	
	6-Junction Droplet Chip	Glass device	-The device consists of 6 separate flow-focusing junctions.<br>-Excellent chemical compatibility.<br>-Hydrophilic, hydrophilic and, fluorophilic coating options.	Designed for the parallel generation of 20 µm - 60 µm oil-in-water droplets.	
Microfluidic ChipShop	Fluidic 162	Topas® COC (Cyclic olefin copolymer) or Polycarbonate PC	-One channel device.<br>-Lid thickness 140 or 170 µm without surface treatment.	Designed for droplet generation on chip.	[2]
	Fluidic 537		-The flow-focusing device consists of 4 identical droplet generation units with a 38 µm nozzle size.		
	Fluidic 912		-The flow-focusing device consists of 8 identical droplet generation units with a channel dimension of 80 µm at the droplet formation region.		
	Fluidic 536		-The device consists of double-cross geometry with 37 µm nozzle size and 3 droplet generator units.	Designed for W/W/O double emulsion generation, allowing cell and particle encapsulation.	
	Fluidic 488		-The device consists of a combination of multiple T-junctions.<br>-Storage module for capturing droplets.<br>-Double cross nozzle type (74 µm).	Designed for generating single or multiple emulsions and storage.	

	Fluidic 285		-The multi-channel device presents various channels with different nozzle (50; 70; 80; 100 μm).<br>-The main channel, as well as the entrance channel, vary in diameter, enabling a large set of experiments.	Designed for generating droplets with different volumes.	
	Fluidic 1196	Glass microfluidic device	-Several parallel microchannels in one unit.<br>-Lid thickness of 250 μm -Channel dimensions 20 μm	Designed for droplet generation on chip.	

Table S2. Technological approaches and properties of the delivery systems based on food-grade emulsions assembled by microfluidic techniques.

Emulsion type-Delivery system	Aqueous phase	Oil phase	Process conditions	Bioactive	Delivery system characterization	Microfluidic device type	Reference
O/W- Emulsion	Modified lecithin (ML), whey protein isolate (WPI), or Tween 20 (0.05-3 wt%)	Medium chain triacylglycerol oil	Q_d= 0.1-10 mL/h<br>Q_c= 100-500 mL/h	Fucoxanthin extract (0-4 wt%)	Size= 30.3 ± 0.2 µm (ML), 32.3 ± 0.1 µm (WPI) and 29.0 ± 0.8 µm (Tween 20)<br>DI= 0.5<br>EE= 100%(ML)<br>90.7% (WPI)<br>50.4% (Tween 20)	Silicon 24 × 24 mm^2 microchannels array chip (Model: WMS 11–1; EP. Tech Co., Ltd., Hitachi, Japan)<br>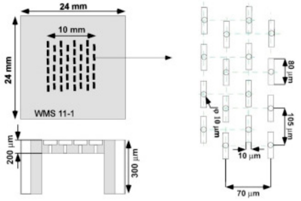 	[3]
	Tween 20 (1% w/w), sodium salt of colic acid (Na-cholate), decaglycerol monolaurate, polyglyceryl-5-		J_d= 10-300 L/m.h	Quercetin (0.1-0.6 mg/mL)	Size= 28-29 µm<br>DI < 0.21<br>EE= 80 % (4 ºC) and 70 % (25 ºC)	Silicon 24 × 24 mm^2 microchannels array chip (Model WMS 1-2; EP. Tech Co., Ltd., Hitachi, Japan)<br>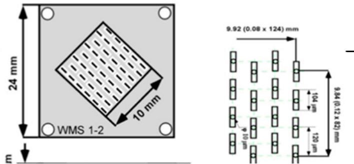 	[4]

	laurate (Sunsoft A-12E), or bovine serum albumin (BSA) (1% w/w)						
	Decaglycerol monolaurate (ML-750) or Tween 20 (1% w/w)		Q_d= 1-14 mL/h	β-sitosterol (0.5-4.0% w/w) and γ-oryzanol (0.5-4.0% w/w)	Size= 26-28 µm<br>DI < 0.20<br>EE over 80 %	Silicon 24 × 24 mm^2 microchannels array chip (Model: WMS 11–1; EP. Tech Co., Ltd., Hitachi, Japan)	[5]
	Sodium dodecyl sulfate (SDS), decaglycerol monolaurate (ML-750), decaglycerol monooleate (MO-7S), sodium casinate (Na-Cs), and modified lecithin (ML) (1% w/w)		Q_d= 0.25-5 mL/h	AstaReal® (AR, astaxanthin purity 20%), Zanthin® (ZA, astaxanthin purity 10%), and Astaxanthin >97% (Sigma-Aldrich) (1-5% w/w)	Size= 35-37 µm<br>DI < 0.25<br>EE over 98 %	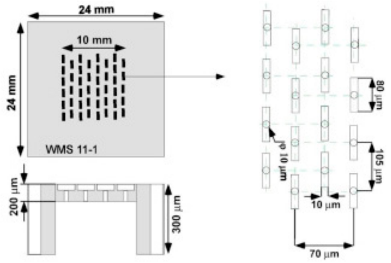 	[5]
O/W-Microgel	Sodium alginate (1 wt%), gelatin (5 wt%), and EDTA-Ca (2 wt%)	Tert-butyl hydroquinone (TBHQ)	Q_d= 1-2 mL/h<br>Q_c= 10-20 mL/h	Vitamin A (weight ratio 4:1 vitamin A/TBHQ)	Size= 246 µm<br>DI= 0.96%<br>EE= not specified<br>CR= 75% over 2h	Photosensitive resin microfluidic device made using microscale 3D printing equipment<br>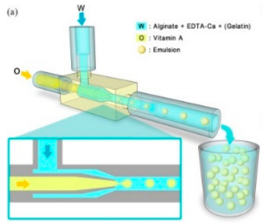 	[6]
	Poly (vinyl alcohol) (PVA) (0.25% w/v)	PLGA 7525A dissolved in dichloromethane (DCM)	-	Finasteride (28 mg, monthly doses)	Size around 30 µm<br>DI= 23-28%<br>EE= not specified<br>CR= an initial burst, a moderate release, and then a plateau.	IVL-PPF Microsphere® microfluidic device<br>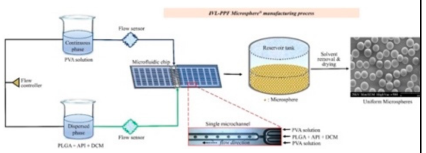	[7]
		PLGA 5050A (PURASORB® PDLG 5002A),	P_d= 1,100 mbar		Size= 40 µm (PLGA 7502A or 5002A) and		[7]

		PLGA 7525A (PURASORB® PDLG 7502A), and PLA02A (PURASORB® PDL 02A) dissolved in dichloromethane (DCM)	P_c= 2,200 mbar		around 30 µm (PLGA/PLA02A)<br>DI= 0.28 and 0.16<br>EE > 96.5%		
O/W-Nanoparticle	D1: acetone or methanol<br>D2: deionized water	3M fluorinated™ Fluid (FC-40)	Q_c= 10 to 250 µL/min.<br>Q_d= fixed at 50 µL/min.	Itraconazole (ITZ) nanoparticles (1 mg/mL)	Size= the volume was calculated and range from 1 to 6 µL.<br>EE= encapsulation was confirmed by the increase of particle size.	316 stainless steel metal T-junction devices<br>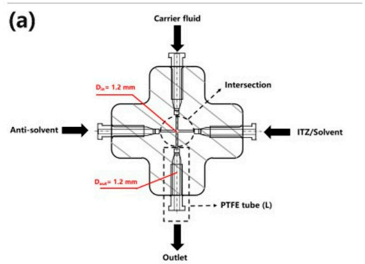 	[8]
	Poly (vinyl alcohol) (PVA; 1% w/v) solution	PLGA (13.4 mg/mL) in acetonitrile (ACN)	Total flow rate: 2-12 mL/min<br>Flow rate ratio= 2:1 to 10:1, aqueous:oil phases	Rutin (10 mg/mL)	Size= 123.4 nm<br>DI= 0.16 ± 0.005<br>EE= 34 ± 2%	NanoAssemblr® Benchtop Device<br>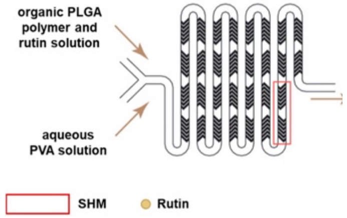 	[9]
	Water	Tween 20 dissolved in ethyl acetate	P_c= 4.20 bar<br>P_d= 3.81 bar<br>For the smallest particle size	Fenofibrate (0.5 %wt)	Size < 1 µm<br>DI= not specified	Glass microfluidic device<br>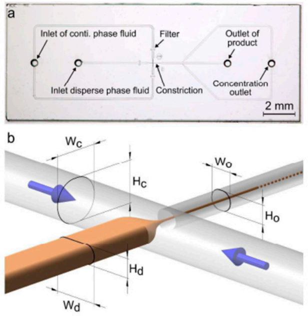 	[10]
O/W/O-	*Middle phase:* an aqueous solution	*Outer phase:* soybean oil,	Q_i= 2.2 µL/h.	Thyme essential oil and lavender	Size= 182-342 µm<br>DI= 2.4-2.8%	Glass microfluidic device	[11]

Core-shell microcapsule	containing $CaCl_2$ (100 × 10^{-3} M), disodium-EDTA (100 × 10^{-3} M) and sodium alginate (2.0%; pH 7.0)	acetic acid (5% w/v) and PGPR (5.0% w/v)<br>*Inner phase:* soybean oil, benzyl benzoate (1:1), and PGPR (2.0% w/v)	Q_m= 5.0 µL/h.<br>Q_o= 68.1 µL/h	essential oil (33.33% v/v)	EE= not specified	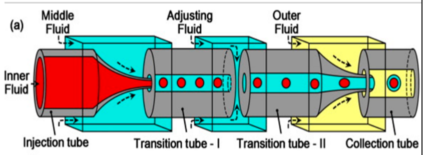	
W/O/W- Giant liposome	*Outer phase:* poly (vinyl alcohol) (PVA; 10 % w/v)<br>*Inner phase:* PVA (1% w/v) and dextran (9% w/v)	*Middle phase:* soybean lecithin (0.5% w/v) in the following organic solvent mixtures (1:1.8 v/v): chloroform/hexane; ethyl acetate/hexane or ethyl acetate/pentane.	Q_i= 1000 µl/h<br>Q_m= 1000 µl/h<br>Q_o= 3,000-12,000 µl/h.	β-carotene (0.125% w/v)	Size= 100-180 µm<br>DI= 3-6 %<br>EE= not specified	Glass microfluidic device<br>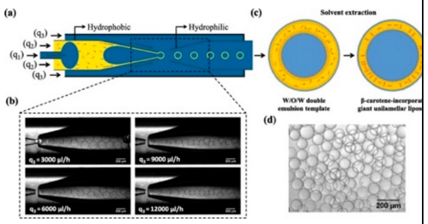	[12]
W/O-Microgel	Pectin solution (0.5 and 1% w/w)	Acetic acid and $CaCO_3$ in rapeseed oil	Q_d= 1 mL/h<br>Q_c= 9 mL/h	Silver and gold nanoparticles (5.5 nm; 1.5 mg/mL)	The microgel was able to encapsulate nanoparticles	Polycarbonate (PC) microfluidic device<br>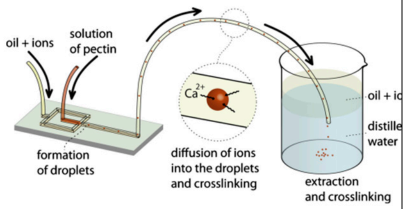	[13]
	Gellan gum (0.10–0.30% w/w) and *Jabuticaba* extract (diluted in 0.025 M potassium chloride pH 1 and 0.4 M sodium acetate pH 4.5)	PGPR (4% w/w) and calcium acetate (1% w/w) added to the soybean oil	Q_d= 2-30 mL/h<br>Q_c= 150-250 mL/h	*Jabuticaba* extract (10-30% v/v)	Size= 185-342 µm<br>DI= 0.016 and 0.086	Glass microfluidic device<br>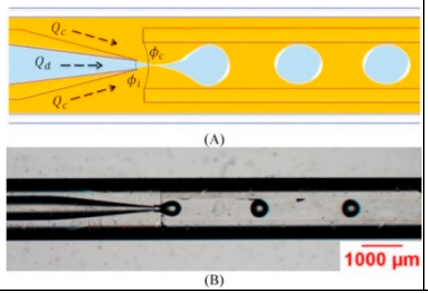	[14]

	1: Dextran or protein aqueous solutions<br>2: Alginate solution (2% wt%) containing $CaCO_3$ (200 mM). poly(ethyleneimine) (PEI; 0.3 wt%) or chitosan (1% v/v in acetic acid) coating	3: Mineral oil with Span 80 (3 wt%), used as oil phase A<br>4: Mineral oil with Span 80 and acetic acid (0.2% v/v) used as oil phase B	Q_1= 15 µL/min<br>Q_2= 0.5 µL/min<br>Q_3= 10 µL/min<br>Q_4= 14 µL/min	Ovalbumin	Size around 100 µm<br>DI= not specified<br>EE= 88% (PEI coating) and 80% (chitosan coating)	Polydimethylsiloxane (PDMS) microfluidic device (four inlets and one outlet)<br>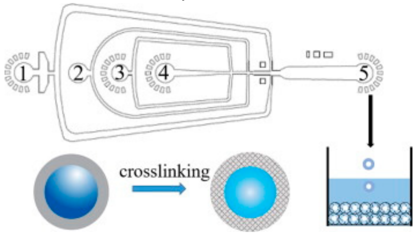	[15]
	Alginate (2% w/v) and recombinant proteins in water<br>*Collection bath:* chitosan (0.5%) and $CaCl_2$ (0.1%)	Span 80 in mineral oil	Q_d= 10 µl/h<br>Q_c= 50 µl/h	eGFP+AvrA nanoparticles (2% w/v)	Size= 339 ± 52 µm<br>DI= 0.352 ± 0.153<br>EE= not specified<br>CR= 70 % of the encapsulated was released after 240 min	Polydimethylsiloxane (PDMS) microfluidic device	[16]
	Pectin (1 wt%)<br>Sheath flow: $CaCl_2$ solution (1 wt%)	Mineral oil solution containing quercetin nanoparticles and retinyl palmitate, and Tween 80 (1 wt%)	Q_d= 0.5 µL/min<br>Q_c= 4.0 µL/min<br>Q_{sheath}= 30 µL/min	Quercetin nanoparticles (0.02 wt%) and retinyl palmitate (0.2 wt%)	Size around 67.3-93.1 µm<br>DI= not specified<br>EE= not specified, encapsulation confirmed by fluorescence	Polydimethylsiloxane (PDMS) microfluidic device<br>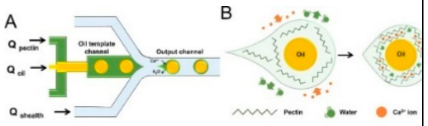	[17]
W/O/W-Emulsion	*Inner phase:* phosphate buffer solution (100 mmol/L) containing betanin and D-glucose (1% w/w)<br>*Outer phase:* Tween 20 in water	*Middle phase:* soybean oil and tetraglycerin monolaurate condensed ricinoleic acid ester (CR-310)	J_d= 5-100 L/m.h	E162, (mixture of beetroot extract and maltodextrin; 0.4% w/w betanin, TCI/ABCr), betanin, and spray dried beetroot juice) (0.1-1.0% w/w)	Size < 50 µm<br>DI < 0.26 ± 0.01	Silicon 24 × 24 mm^2 microchannels array chip (Model: WMS 11–1; EP. Tech Co., Ltd., Hitachi, Japan)<br>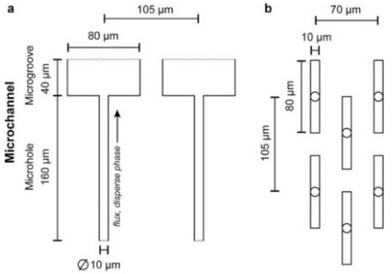	[18]

W/O/W- Solid lipid microparticle	*Outer phase:* water and polyvinyl alcohol (PVA; 10% (w/v)<br>*Inner phase:* Ascorbic acid solution with or without $CaCl_2$, and chitosan.	*Middle phase:* palm fat oil	Q_i= 3,000 and 1,000 µL/h.<br>Q_m= 2,500 and 3,000 µL/h.<br>Q_o= 14,000 and 12,000 µL/h	Ascorbic acid (3-20% w/w)	Size= 195- 342 µm<br>DI= 73-95 %<br>EE= 73-95 %<br>CR decreased around 50% after 30 days	Glass microfluidic device<br>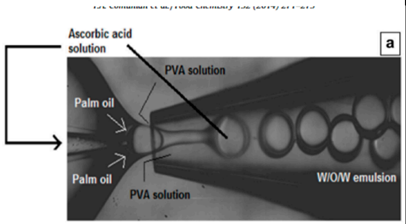	[19]
W/O/W-Microcapsule	*Outer phase:* aqueous solution and PVA<br>*Inner phase:* aqueous mesoporous silica nanoparticles (MSN) + Rhodamine B (RB) solution	*Middle phase:* PLGA oil	Q_i= 1 mL/h.<br>Q_m= 2 mL/h.<br>Q_o= 4 mL/h	Mesopouros silica nanoparticles (MSN; 40% w/v) + Rhodamine B (RB; 10-500 mg/L)	Size= 119 nm (MSN) and 56 µm (PLGA-MSN)<br>DI= 4.91%<br>EE around 88% for PLGA-MSN and 95% for PLGA<br>CR around 96% after 120 days for PLGA-MSN and<br>97% after 120 days for PLGA	Polymethyl methacrylate (PMMA) microfluidic device<br>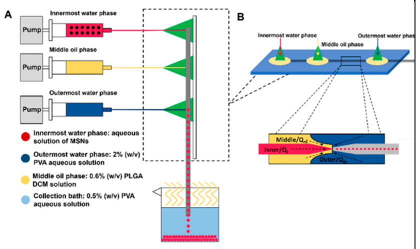	[20]
	Outer phase: Poly (vinyl alcohol) (PVA) aqueous solution (2 wt%)<br>*Inner phase:* Poly (vinyl alcohol) (PVA) aqueous solution	*Middle phase:* PLGA (0.6 wt%) in dichloromethane)	Q_i= 1000 µL/min Q_m= 2000 µL/min<br>Q_o= 4000 µL/h	2-[[(4-phenoxyphenyl) sulfonyl]methyl] -thiirane (SB-3CT; 0.5 mg/mL)	Size= 35-65 µm<br>DI= 3%<br>EE= 99%	Glass microfluidic device<br>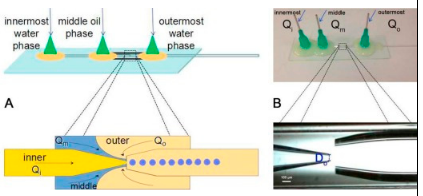	[21]
	Middle phase: poly (N-isopropyl acry-lamide) (PNIPAM)	*Inner phase:* soybean oil solution added with camptothecin (0.4 mg/mL)	Q_o= 1600-2400 µL/min<br>Q_m= 130-210 µL/min<br>Q_i= 20-100 µL/min	Doxorubicin hydrochloride and camptothecin (2 mg/mL)	Size= 600-1000 µm<br>DI= not specified<br>EE= not specified<br>CR= burst process	Glass microfluidic device	[22]

		Outer phase: soybean oil and PGPR					

*Q_c: continuous phase flow rate; Q_d: disperse phase flow rate; Q_i: inner phase flow rate; Q_m: middle phase flow rate; Q_o: outer phase flow rate; J_d: disperse phase flux; P_d: disperse phase pressure; P_c: continuous phase pressure; Tween 20: polyoxyethylene (20) sorbitan monolaurate; EDTA-Ca: ethylenediaminetetraacetic acid calcium disodium salt hydrate; PGPR: polirricinoleato de poliglicerol; $CaCl_2$: calcium chloride; $CaCO_3$: calcium carbonate; PLGA: poly-lactic-co-glycolic acid; DI: dispersity index; EE: encapsulation efficiency; CR: compound release.

Table S3. Trends in droplet-based microfluidics approaches for fabricating delivery systems based on emulsions.

Emulsion type	Continuous phase	Disperse phase	Surfactant	Microfluidic device type	Process conditions	Microfluidic device design	Reference
O/W emulsion	Aqueous surfactant solution	Palm oil	Span 20	Combination of ultrasound and silicon/epoxy microchannel	Q_d and Q_c= 4 mL/min	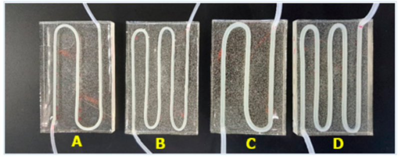	[23]
	Xanthan gum or poloxamer in water	Sunflower oil	Tween 80 or Precirol™	Microsystem at High Throughput (MHT)	Q_d= 20.8-95.2 mL/min Q_c= 500 mL/min.	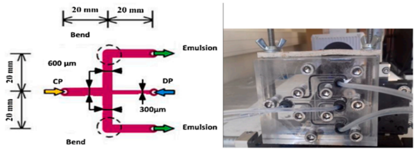	[24]
	Argan by-products extract	Soybean oil	Tween 80	Silicon	Q_d= 2 mL/h	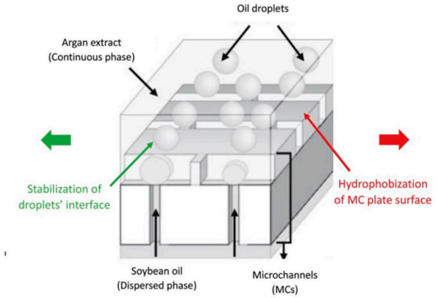	[25]
	Mineral oil and glutaraldehyde (5 %wt)	Chitosan (2 %wt) in hydrochloric acid (1 %wt)	Span 80 (3 %wt)	PDMS microfluidic device	Q_d and Q_c = 100 to 500 µL/h	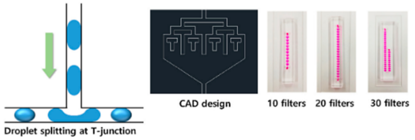	[26]

						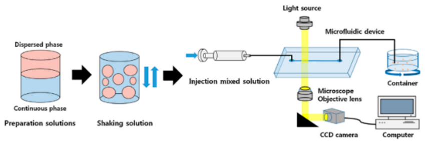	
	Silicon oil and surfactant	Deionized water	Span 80 (1% w/v)	Photosensitive resin Gr		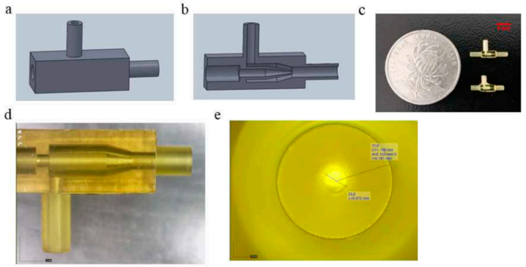	[27]
	Water and PEG 2000	Sunflower oil	Tween 20	Glass microfluidic device	Q_d and Q_c= 20 to 280 µL/min	-	[28]
W/O emulsion	Sunflower oil and surfactant	Whey protein isolated (WPI)	PGPR	Glass microfluidic device	Pressure ranged from 0-200 mbar for both phases	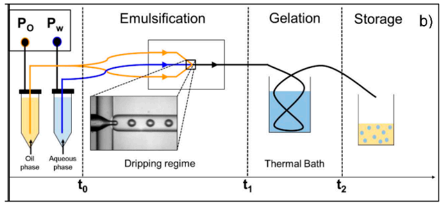	[29]
	Fluorocarbon oil (HFE 7500) and surfactant	PPGDA:Dextran (20% w/w) Dextran and PPGDA:PPGA (1:3 in 300 µL of ethanol)	Fluorosurfactant 008 (2% w/w)	PDMS microfluidic device	Q_d= 60 µL/h for PPGDA and 30 µL/h for dextran	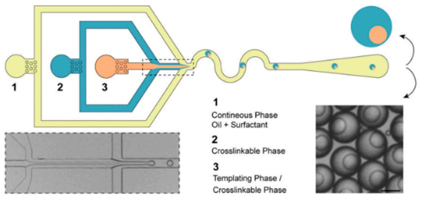	[30]
	Deionized water and surfactant	Mixture of mineral oil and heptane (50:50).	PVA (2% w/w).	Photosensitive resin Gr	Q_d and Q_c= 100 to 500 µL/h	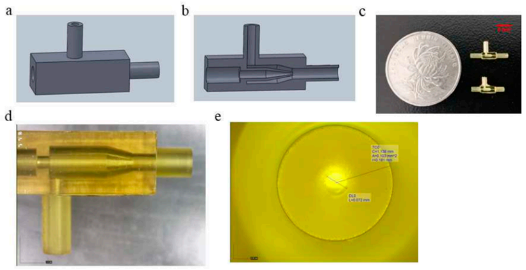	[27]

W/O/W emulsion	*Outer phase:* glycerol (5% w/v)	*Inner phase:* glycerol (50% w/v) in water<br>*Middle phase*: middle chain triglyceride (MCT)	PGPR in MCT	Glass microfluidic device	Q_i= 1-5 mL/h<br>Q_m= 4-14 mL/h<br>Q_o= 50-400 mL/h	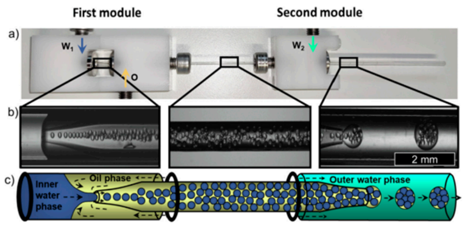	[31]
	Outer phase: glycerol (25% w/w) in distilled water	*Inner phase:* Na-alginate (1.5 %wt)<br>*Middle phase*: silicon oil	Span 80 (0.25% w/w) in middle phase.<br>PVA (5% w/w) in outer phase	PDMS microfluidic device	Q_i = 50 µL/h.<br>Q_m= 500 µL/h<br>Q_o=: 20000 - 100000 µL/h		[32]
	Outer phase: aqueous surfactant solution	*Middle phase:* phenylmethyl silicone oil and silicone resin RSN-0749<br>*Inner phase:* glycerol, PVA, and water	PVA (5 wt%) in outer and inner phases.	Glass microfluidic device	Q_i= 150 mL/h<br>Q_m= 150-1750 mL/h<br>Q_o= 1 mL/h	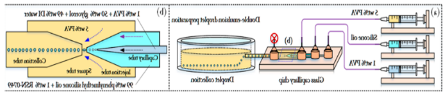	[33]
	Outer phase: glycerol (40 wt%) and surfactant	*Inner phase:* glycerol (5 %wt) and surfactant<br>*Middle phase:* XIA-METERÒ RSN-0749 resin in Dow Corning ® 200 fluid (2 wt%)	Hydrophilic: PVA<br>Lipophilic: XIAMETER® RSN-074	Glass microfluidic device		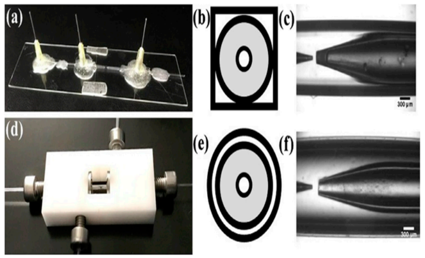	[34]
O/W/O emulsion	*Outer phase 1 and 2:* oil solution containing PGPR (4% w/v)	*Inner phase:* poly(ethylene glycol) diacrylate (PEGDA; 50 %wt), 2-Hydroxy-methylpropiophenone (HMPP; 5 %wt), and surfactant	Pluronic ® F 127	Glass microfluidic device	Q_i= 100-900 µL/h<br>Q_{o1}= 300 and 200 µL/h<br>Q_{o2}= 500-800 µL/h	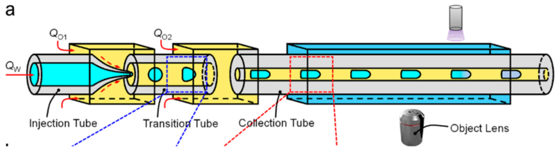	[35]

<table>
<tr><td></td><td></td><td></td><td></td><td></td><td></td><td></td><td></td></tr>
<tr><td></td><td>Outer phase: dimethicone</td><td>Inner phase: silicone oil.
Middle phase: surfactant</td><td>PVA (2 % w/w) in the middle phase</td><td>Photosensitive resin Gr and HIT</td><td>Q_i= Q_m= 2 mL/h
Q_c= 5 mL/h</td><td>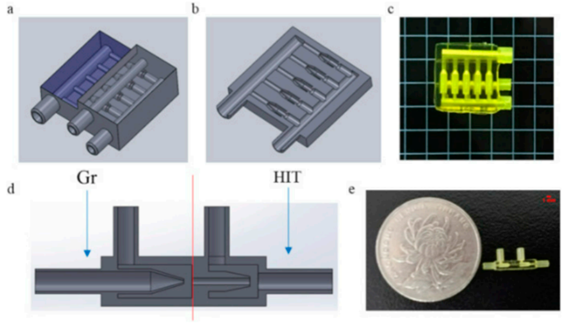
</td><td>[27]</td></tr>
<tr><td></td><td>Outer phase: sunflower oil and glacial acetic acid (0.1% w/w)</td><td>O/W emulsion prepared by ultrasound: Na-alginate (1% (w/w)) and $CaCO_3$ (0.1825 g/g of Na-alginate), cellulose nanocrystal and sunflower oil</td><td>PGPR (4 wt%)</td><td>Glass microfluidic device</td><td>Q_c= 3000 - 15000 µL/h.
Q_d= 600-3000 µL/h</td><td>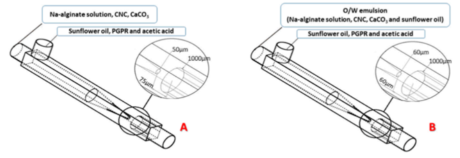
</td><td>[36]</td></tr>
<tr><td></td><td>Outer phase: silicone oil</td><td>Inner phase: silicone oil.
Middle phase: glycerol (25% (w/w))</td><td>Span 80 (0.25% w/w) in the inner phase.
PVA (10 % w/w) and in the middle phase.
Span 80 (2.5% w/w) in the outer phase</td><td>PDMS microfluidic device</td><td>Q_i= 50 µL/h.
Q_m= 500 µL/h
Q_o= 20,000 - 100,000 µL/h</td><td>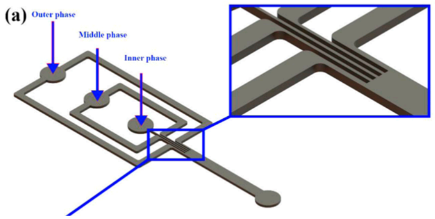
</td><td>[32]</td></tr>
<tr><td></td><td>Outer phase: orange oil</td><td>Inner phase: middle chain triglyceride (MCT).
Middle phase: PVA (1% w/v) in water</td><td>PVA in water and PGPR in MCT</td><td>Glass microfluidic device</td><td>Q_o=50-400 mL/h
Q_m= 4-14 mL/h
Q_i=1-5 mL/h</td><td>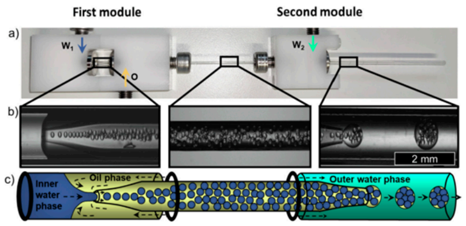
</td><td>[31]</td></tr>
</table>

* PVA: polyvinyl alcohol; PGPR: polyglycerol polyricinoleate; Span 20: sorbitan monooleate; PDMS: polydimethylsiloxane.

References

1. Dolomite Microfluidics, 2022 Dolomite Microfluidics, URL: https://www.dolomite-microfluidics.com/, 29 January 2022.
2. microfluidic ChipShop, 2022 Microfluidic ChipShop, URL: https://www.microfluidic-chipshop.com/, 29 January 2022.
3. Ma, Z.; Zhao, Y.; Khalid, N.; Shu, G.; Neves, M.A.; Kobayashi, I.; Nakajima, M. Comparative Study of Oil-in-Water Emulsions Encapsulating Fucoxanthin Formulated by Microchannel Emulsification and High-Pressure Homogenization. *Food Hydrocoll.* **2020**, *108*, 105977, doi:10.1016/J.FOODHYD.2020.105977.
4. Khalid, N.; Kobayashi, I.; Neves, M.A.; Uemura, K.; Nakajima, M.; Nabetani, H. Microchannel Emulsification Study on Formulation and Stability Characterization of Monodisperse Oil-in-Water Emulsions Encapsulating Quercetin. *Food Chem.* **2016**, *212*, 27–34, doi:10.1016/J.FOODCHEM.2016.05.154.
5. Khalid, N.; Shu, G.; Kobayashi, I.; Nakajima, M.; Barrow, C.J. Formulation and Characterization of Monodisperse O/W Emulsions Encapsulating Astaxanthin Extracts Using Microchannel Emulsification: Insights of Formulation and Stability Evaluation. *Colloids Surfaces B Biointerfaces* **2017**, *157*, 355–365, doi:10.1016/J.COLSURFB.2017.06.003.
6. Zhang, J.; Zhang, R.; Zhang, Y.; Pan, Y.; Shum, H.C.; Jiang, Z. Alginate-Gelatin Emulsion Droplets for Encapsulation of Vitamin A by 3D Printed Microfluidics. *Particuology* **2021**, doi:10.1016/J.PARTIC.2021.09.004.
7. Kim, J.H.; Na, J.; Bak, D.H.; Lee, B.C.; Lee, E.; Choi, M.J.; Ryu, C.H.; Lee, S.; Mun, S.K.; Park, B.C.; et al. Development of Finasteride Polymer Microspheres for Systemic Application in Androgenic Alopecia. *Int. J. Mol. Med.* **2019**, *43*, 2409–2419, doi:10.3892/IJMM.2019.4149/HTML.
8. Kim, S.; Wang, H.; Yan, L.; Zhang, X.; Cheng, Y. Continuous Preparation of Itraconazole Nanoparticles Using Droplet-Based Microreactor. *Chem. Eng. J.* **2020**, *393*, 124721, doi:10.1016/J.CEJ.2020.124721.
9. Vu, H.T.H.; Streck, S.; Hook, S.M.; McDowell, A. Utilization of Microfluidics for the Preparation of Polymeric Nanoparticles for the Antioxidant Rutin: A Comparison with Bulk Production. *Pharm. Nanotechnol.* **2019**, *7*, 469–483, doi:10.2174/2211738507666191019141049.
10. Lorenz, T.; Bojko, S.; Bunjes, H.; Dietzel, A. An Inert 3D Emulsification Device for Individual Precipitation and Concentration of Amorphous Drug Nanoparticles. *Lab Chip* **2018**, *18*, 627–638, doi:10.1039/C7LC01313B.
11. Mou, C.L.; Deng, Q.Z.; Hu, J.X.; Wang, L.Y.; Deng, H.B.; Xiao, G.; Zhan, Y. Controllable Preparation of Monodisperse Alginate Microcapsules with Oil Cores. *J. Colloid Interface Sci.* **2020**, *569*, 307–319, doi:10.1016/J.JCIS.2020.02.095.
12. Michelon, M.; Huang, Y.; de la Torre, L.G.; Weitz, D.A.; Cunha, R.L. Single-Step Microfluidic Production of W/O/W Double Emulsions as Templates for β-Carotene-Loaded Giant Liposomes Formation. *Chem. Eng. J.* **2019**, *366*, 27–32, doi:10.1016/J.CEJ.2019.02.021.
13. Ogończyk, D.; Siek, M.; Garstecki, P. Microfluidic Formulation of Pectin Microbeads for Encapsulation and Controlled Release of Nanoparticles. *Biomicrofluidics* **2011**, *5*, 013405, doi:10.1063/1.3569944.
14. Santos, T.P.; Costa, A.L.R.; Michelon, M.; Costa, L.P.; Cunha, R.L. Development of a Microfluidic Route for the Formation of Gellan-Based Microgels

Incorporating Jabuticaba (Myrciaria Cauliflora) Extract. *J. Food Eng.* **2020**, *276*, 109884, doi:10.1016/J.JFOODENG.2019.109884.

15. Yu, L.; Sun, Q.; Hui, Y.; Seth, A.; Petrovsky, N.; Zhao, C.X. Microfluidic Formation of Core-Shell Alginate Microparticles for Protein Encapsulation and Controlled Release. *J. Colloid Interface Sci.* **2019**, *539*, 497–503, doi:10.1016/J.JCIS.2018.12.075.
16. Ling, K.; Wu, H.; Neish, A.S.; Champion, J.A. Alginate/Chitosan Microparticles for Gastric Passage and Intestinal Release of Therapeutic Protein Nanoparticles. *J. Control. Release* **2019**, *295*, 174–186, doi:10.1016/J.JCONREL.2018.12.017.
17. Noh, J.; Kim, J.; Kim, J.S.; Chung, Y.S.; Chang, S.T.; Park, J. Microencapsulation by Pectin for Multi-Components Carriers Bearing Both Hydrophobic and Hydrophilic Active Agents. *Carbohydr. Polym.* **2018**, *182*, 172–179, doi:10.1016/J.CARBPOL.2017.11.026.
18. Pagano, A.P.E.; Khalid, N.; Kobayashi, I.; Nakajima, M.; Neves, M.A.; Bastos, E.L. Microencapsulation of Betanin in Monodisperse W/O/W Emulsions. *Food Res. Int.* **2018**, *109*, 489–496, doi:10.1016/J.FOODRES.2018.04.053.
19. Comunian, T.A.; Abbaspourrad, A.; Favaro-Trindade, C.S.; Weitz, D.A. Fabrication of Solid Lipid Microcapsules Containing Ascorbic Acid Using a Microfluidic Technique. *Food Chem.* **2014**, *152*, 271–275, doi:10.1016/J.FOODCHEM.2013.11.149.
20. Zhou, J.; Zhai, Y.; Xu, J.; Zhou, T.; Cen, L. Microfluidic Preparation of PLGA Composite Microspheres with Mesoporous Silica Nanoparticles for Finely Manipulated Drug Release. *Int. J. Pharm.* **2021**, *593*, 120173, doi:10.1016/J.IJPHARM.2020.120173.
21. Chen, H.; Jia, F.; Zhu, C.; Xu, J.; Hua, X.; Xi, Z.; Shen, L.; Zhao, S.; Cen, L. Controllable Preparation of SB-3CT Loaded PLGA Microcapsules for Traumatic-Brain-Injury Pharmaco-Therapy. *Chem. Eng. J.* **2018**, *339*, 346–358, doi:10.1016/J.CEJ.2018.01.140.
22. Chen, Z.; Song, S.; Ma, J.; Ling, S. Da; Wang, Y.D.; Kong, T.T.; Xu, J.H. Fabrication of Magnetic Core/Shell Hydrogels via Microfluidics for Controlled Drug Delivery. *Chem. Eng. Sci.* **2022**, *248*, 117216, doi:10.1016/J.CES.2021.117216.
23. Manickam, S.; Sivakumar, K.; Pang, C.H. Investigations on the Generation of Oil-in-Water (O/W) Nanoemulsions through the Combination of Ultrasound and Microchannel. *Ultrason. Sonochem.* **2020**, *69*, 105258, doi:10.1016/J.ULTSONCH.2020.105258.
24. Nehme, R.; Blel, W.; Montillet, A.; Bellettre, J.; Marchal, L. Production of Oil in Water Emulsions in Microchannels at High Throughput: Evaluation of Emulsions in View of Cosmetic, Nutraceutical or Pharmaceutical Applications. *Chem. Eng. Process. - Process Intensif.* **2021**, *161*, 108301, doi:10.1016/J.CEP.2021.108301.
25. Taarji, N.; Vodo, S.; Bouhoute, M.; Khalid, N.; Hafidi, A.; Kobayashi, I.; Neves, M.A.; Isoda, H.; Nakajima, M. Preparation of Monodisperse O/W Emulsions Using a Crude Surface-Active Extract from Argan by-Products in Microchannel Emulsification. *Colloids Surfaces A Physicochem. Eng. Asp.* **2020**, *585*, 124050, doi:10.1016/J.COLSURFA.2019.124050.
26. Kim, C.M.; Choi, H.J.; Park, E.J.; Kim, G.M. Repeated Geometrical T-Junction Breakup Microfluidic Filter Device by Injection of Premixed Emulsion for Microdroplet Production. *J. Ind. Eng. Chem.* **2020**, *81*, 81–87, doi:10.1016/J.JIEC.2019.08.055.
27. Zhang, J.; Xu, W.; Xu, F.; Lu, W.; Hu, L.; Zhou, J.; Zhang, C.; Jiang, Z. Microfluidic Droplet Formation in Co-Flow Devices Fabricated by Micro 3D Printing. *J. Food Eng.* **2021**, *290*, 110212, doi:10.1016/J.JFOODENG.2020.110212.

28. Jurinjak Tušek, A.; Jurina, T.; Čulo, I.; Valinger, D.; Gajdoš Kljusurić, J.; Benković, M. Application of NIRs Coupled with PLS and ANN Modelling to Predict Average Droplet Size in Oil-in-Water Emulsions Prepared with Different Microfluidic Devices. *Spectrochim. Acta Part A Mol. Biomol. Spectrosc.* **2022**, *270*, 120860, doi:10.1016/J.SAA.2022.120860.

29. Lacroix, A.; Hayert, M.; Bosc, V.; Menut, P. Batch versus Microfluidic Emulsification Processes to Produce Whey Protein Microgel Beads from Thermal or Acidic Gelation. *J. Food Eng.* **2022**, *312*, 110738, doi:10.1016/J.JFOODENG.2021.110738.

30. Keller, S.; Dekkers, R.; Hu, G.X.; Tollemeto, M.; Morosini, M.; Keskin, A.; Wilson, D.A. A Simple Microfluidic Tool to Design Anisotropic Microgels. *React. Funct. Polym.* **2021**, *167*, 105012, doi:10.1016/J.REACTFUNCTPOLYM.2021.105012.

31. Leister, N.; Vladisavljević, G.T.; Karbstein, H.P. Novel Glass Capillary Microfluidic Devices for the Flexible and Simple Production of Multi-Cored Double Emulsions. *J. Colloid Interface Sci.* **2022**, *611*, 451–461, doi:10.1016/J.JCIS.2021.12.094.

32. Sattari, A.; Hanafizadeh, P.; Keshtiban, M.M. Microfluidic Preparation of Double Emulsions Using a High Aspect Ratio Double Co-Flow Device. *Colloids Surfaces A Physicochem. Eng. Asp.* **2021**, *628*, 127297, doi:10.1016/J.COLSURFA.2021.127297.

33. Zhang, K.; Ren, Y.; Jiang, T.; Jiang, H. Thermal Field-Actuated Multifunctional Double-Emulsion Droplet Carriers: On-Demand Migration, Core Release and Released Particle Focusing. *Chem. Eng. J.* **2022**, *431*, 134200, doi:10.1016/J.CEJ.2021.134200.

34. Bandulasena, M. V.; Vladisavljević, G.T.; Benyahia, B. Versatile Reconfigurable Glass Capillary Microfluidic Devices with Lego® Inspired Blocks for Drop Generation and Micromixing. *J. Colloid Interface Sci.* **2019**, *542*, 23–32, doi:10.1016/J.JCIS.2019.01.119.

35. Cai, Q.W.; Ju, X.J.; Chen, C.; Faraj, Y.; Jia, Z.H.; Hu, J.Q.; Xie, R.; Wang, W.; Liu, Z.; Chu, L.Y. Fabrication and Flow Characteristics of Monodisperse Bullet-Shaped Microparticles with Controllable Structures. *Chem. Eng. J.* **2019**, *370*, 925–937, doi:10.1016/J.CEJ.2019.03.221.

36. Dias Meirelles, A.A.; Rodrigues Costa, A.L.; Michelon, M.; Viganó, J.; Carvalho, M.S.; Cunha, R.L. Microfluidic Approach to Produce Emulsion-Filled Alginate Microgels. *J. Food Eng.* **2022**, *315*, 110812, doi:10.1016/J.JFOODENG.2021.110812.
